# Supplementary material for: On an algorithmic definition for the components of the minimal cell
Source: PLoS One. 2018 Jun 1;13(6):e0198222. doi: 10.1371/journal.pone.0198222 (PMC5983409; doi:10.1371/journal.pone.0198222)
Supplement: S1 Text — Additional details and discussion of the C2E algorithm applied to the cases of the RNA polymerase and streptomycin SIs. Also demonstrates the functions and data of our R [72] package ‘InterPlay’ (included as ‘S1 Binary’) to work with SIs and determine essential structures. Algorithms are exemplified and explained in detail and plotting of interactome networks is illustrated with the use of the ‘igraph’ [73] R package (see also S2 Text). (PDF) [file pone.0198222.s001.pdf]

S1 Text: Additional text and computational examples for "On an algorithmic definition for the components of the minimal cell"

OCTAVIO MARTÍNEZ
AND M. HUMBERTO REYES-VALDÉS

Abstract. Here we present additional details and discussion of the C2E algorithm applied to the cases of the RNA polymerase and streptomycin synthesis interactomes (SIs). Additionally, we demonstrate the functions and data of our R package 'InterPlay' to work with SI and determine essential structures. The algorithms implemented in the InterPlay package are exemplified and explained in detail, and plotting of interactome networks are illustrated with the use of the 'igraph' R package (Csardi and Nepusz, 2006). The computational examples in this appendix can be followed performing a step by step analysis in R, or read without following the R examples and focusing in the results for a better understanding of the procedures.

Contents

S1-1. The C2E algorithm applied to the RNA polymerase SI 1
S1-2. The C2E algorithm applied to the streptomycin SI 2
S1-3. Implications of the C2E algorithm 4
S1-4. Notes and prerequisites 5
S1-5. Computational representation of synthesis interactomes (SIs) 6
S1-5.1. Latent sets in an SI 7
S1-5.2. The 'condensed to expanded' (C2E) algorithm 8
S1-5.3. Avoiding infinite loops in the search for expanded formulae 12
S1-6. Distinguishing essential cell elements in SIs 13
S1-6.1. Analysis of a more realistic SI 21
S1-7. Visualizing SI relations with graph theory 33
S1-7.1. 'binding' and 'synthesis' networks from the RNA polymerase SI 33
S1-8. Conclusions and perspectives 43
References 43

S1-1. The C2E algorithm applied to the RNA polymerase SI

In Table 2 of the main text we presented the SI for the RNA polymerase, denoted as '`pol`'. Here we present the results of the application of the C2E algorithm to the components of '`pol`', showing that the expanded formulae for all components of '`pol`', including '`pol`' itself are recursive.

Table S1-1 presents the results of applying the C2E algorithm to all the elements of the set

$$\mathbf{S_i} = \{\texttt{2a, a, b, ba, bp, bpb, o, pol, t.a, t.b, t.bp, t.o}\}$$

i.e., to the internal elements defined by the SI in Table 2 of the main text for the RNA polymerase, *pol*.

By inspecting each one of the rows of Table S1-1 we confirm that in all cases the named element (in column 'Name') appears, once or more times in the expanded formula presented in column 'Expanded' of each corresponding row. For example, for '*pol*', the corresponding expanded formula is $<<< g.o, pol >, rib >, << bp, b >, < a, a >>>$ (4th row in Table S1-1), has '*pol*' as operand in the binary operator

Date: May 21, 2018.

Table S1-1. **Results of applying the 'condensed to expanded' (C2E) algorithm for each element of $\mathbf{S_i}$ in the RNA polymerase SI (See Table 2 in main text).**

Name	Condensed	Expanded
bpb	$< bp, b >$	$<<< g.bp, << t.o, rib >, < bpb, 2a >>>, rib >, << g.b, << t.o, rib >, < bpb, 2a >>>, rib >>$
$2a$	$< a, a >$	$<<< g.a, << t.o, rib >, < bpb, 2a >>>, rib >, << g.a, << t.o, rib >, < bpb, 2a >>>, rib >>$
ba	$< bpb, 2a >$	$<<<< g.bp, < o, ba >>, rib >, << g.b, < o, ba >>, rib >>, <<< g.a, < o, ba >>, rib >, << g.a, < o, ba >>, rib >>>$
pol	$< o, ba >$	$<<< g.o, pol >, rib >, << bp, b >, < a, a >>>$
$t.bp$	$< g.bp, pol >$	$< g.bp, <<< g.o, < o, ba >>, rib >, <<< t.bp, rib >, < t.b, rib >>, << t.a, rib >, < t.a, rib >>>>>$
$t.b$	$< g.b, pol >$	$< g.b, <<< g.o, < o, ba >>, rib >, <<< t.bp, rib >, < t.b, rib >>, << t.a, rib >, < t.a, rib >>>>>$
$t.a$	$< g.a, pol >$	$< g.a, <<< g.o, < o, ba >>, rib >, <<< t.bp, rib >, < t.b, rib >>, << t.a, rib >, < t.a, rib >>>>>$
$t.o$	$< g.o, pol >$	$< g.o, << t.o, rib >, < bpb, 2a >>>$
bp	$< t.bp, rib >$	$<< g.bp, <<< g.o, pol >, rib >, << bp, b >, < a, a >>>>, rib >$
b	$< t.b, rib >$	$<< g.b, <<< g.o, pol >, rib >, << bp, b >, < a, a >>>>, rib >$
a	$< t.a, rib >$	$<< g.a, <<< g.o, pol >, rib >, << bp, b >, < a, a >>>>, rib >$
o	$< t.o, rib >$	$<< g.o, < o, ba >>, rib >$

Notes: 'Name' - Name of each one of the elements in the set of internal elements, $\mathbf{S_i}$, of the SI for the RNA polymerase (*pol*; see Table 2 in the main text for the keys of the named elements). 'Condensed' - Condensed formula for the element in 'Name', given by the binary operator; see column 'Binary operator' in Table 2 in the main text. 'Expanded' - Expanded formula found for the corresponding element in 'Name'; the result of the C2E algorithm applied to that structure.

$< g.o, pol >$. Also note that the expanded expression for *pol* given in this row is different to the one that we previously found 'by hand' in equation 3 of the main text. This is due to the fact that the C2E algorithms exits as soon as one term equal to the input name is found in the expanded formula. This do not alter the fact that all the expanded formulae found by the C2E algorithm and presented in Table S1-1 are recursive. The classification of a formula as recursive or non recursive can be automatically computed.

S1-2. The C2E algorithm applied to the streptomycin SI

To give examples of formulae that are not recursive, we present the synthesis of streptomycin, a secondary metabolite exhibiting antibiotic activities, and which is produced by bacteria in the in the genus *Streptomyces* (Schatz et al., 1944). The SI for streptomycin synthesis was summarized from (Flatt and Mahmud, 2007), and it is presented in Table S1-2. Table S1-3 presents the full names for the elements shown in the 'Name' column of Table S1-2.

Note that the set of external elements for the SI in Table S1-2, labeled as $\mathbf{S_e}$ in columns '1st Set' and '2nd Set', gives the '*root*' of the SI and includes apart from the ribosome (*rib*) and the RNA polymerase (*pol*), three secondary metabolites, (28), (34) and (40), corresponding to 'Streptidine 6-phosphate', 'dTDP-L-dihydrostreptose bound to StrH' and 'NDP-N-methyl-L-glucosamine', respectively. In contrast with the essential elements *rib* and *pol*, these secondary metabolites are not essential for cell survival.

Applying the C2E algorithm to the SI in Table S1-2 we obtain the expanded formulae for each one of the internal elements of that interactome. Those formulae are shown in Table S1-4.

TABLE S1-2. **Normalized synthesis Interactome (SI) for streptomycin (STR).**

Name	Binary Operator	1st Set	2nd Set	Type Name	1st Type	2nd Type
$t.StrH$	$\langle g.StrH,\ pol\rangle$	$\mathbf{G}$	$\mathbf{S_e}$	transcript	gene	enzyme
$StrH$	$\langle t.StrH,\ rib\rangle$	$\mathbf{S_i}$	$\mathbf{S_e}$	peptide	transcript	ribosome
$t.DeH$	$\langle g.DeH,\ pol\rangle$	$\mathbf{G}$	$\mathbf{S_e}$	transcript	gene	enzyme
DeH	$\langle t.DeH,\ rib\rangle$	$\mathbf{S_i}$	$\mathbf{S_e}$	enzyme	transcript	ribosome
$t.StrK$	$\langle g.StrK,\ pol\rangle$	$\mathbf{G}$	$\mathbf{S_e}$	transcript	gene	enzyme
$StrK$	$\langle t.StrK,\ rib\rangle$	$\mathbf{S_i}$	$\mathbf{S_e}$	enzyme	transcript	ribosome
$(a.34)$	$\langle (34),\ StrH\rangle$	$\mathbf{S_e}$	$\mathbf{S_i}$	metabolite	metabolite	peptide
(35)	$\langle (a.34),\ (28)\rangle$	$\mathbf{S_i}$	$\mathbf{S_e}$	metabolite	metabolite	metabolite
(41)	$\langle (35),\ (40)\rangle$	$\mathbf{S_i}$	$\mathbf{S_e}$	metabolite	metabolite	metabolite
(42)	$\langle (41),\ DeH\rangle$	$\mathbf{S_i}$	$\mathbf{S_i}$	metabolite	metabolite	enzyme
STR	$\langle (42),\ StrK\rangle$	$\mathbf{S_i}$	$\mathbf{S_i}$	metabolite	metabolite	enzyme

Notes: For meaning of the element names see Table S1-3. Gene names begin with '`g.`' while transcript names begin with '`t.`'. Columns '1st Set' and 'Set 2nd' give the sets in which the first and second operands of 'Binary operator' exist. Columns 'Type Name', '1st Type' and '2nd Type' give the types of elements for column 'Name', and the first and second operands of 'Binary operator', respectively.

TABLE S1-3. **Names of elements involved in the synthesis of the secondary metabolite streptomycin (STR).**

Name	Full Name
rib	Ribosome.
STR	Streptomycin.
pol	RNA polymerase (RNA-pol).
$StrH$	StrH protein predicted to mediate the ligation of (28) with (34).
DeH	Dehydrogenase involved in the step from (42) to STR.
$StrK$	Enzyme streptomycin phosphatase.
(28)	Streptidine 6-phosphate.
(34)	dTDP-L-dihydrostreptose.
$(a.34)$	dTDP-L-dihydrostreptose bound to StrH.
(35)	O-1,4-α-L-dihydrostreptocyl-streptidine 6-phosphate.
(40)	NDP-N-methyl-L-glucosamine.
(41)	Dihydrostreptomycin 6-phosphate.
(42)	Streptomycin 6-phosphate.

Notes: Main elements involved in the synthesis of streptomycin (STR), compiled and simplified from Flatt and Mahmud (2007). Column 'Name' gives the shorten element identifier, as used in the 'Name' column of Table S1-2, while column 'Full name' gives the biochemical designation of the elements.

In the first row of Table S1-4, showing the condensed and expanded formulae for the transcript of the protein StrH, '$t.StrH$', we note that both formulae, condensed and expanded, are identical. This happens in the C2E algorithm when no substitutions are possible to expand the condensed formula. But more interestingly, it can be corroborated that none of the expanded formulae in the 11 rows of Table S1-4 presents within its operands the element being defined, i.e., none of the expanded formula for the synthesis of streptomycin components is recursive. In particular, the last row, showing the expanded formula for

Table S1-4. **Results of applying the 'condensed to expanded' (C2E) algorithm for each element of $\mathbf{S_i}$ in the streptomycin (STR) SI (Table S1-2).**

Name	Condensed	Expanded
$t.StrH$	$< g.StrH,\ pol >$	$< g.StrH, pol >$
$StrH$	$< t.StrH,\ rib >$	$<< g.StrH, pol >, rib >$
$t.DeH$	$< g.DeH,\ pol >$	$< g.DeH, pol >$
DeH	$< t.DeH,\ rib >$	$<< g.DeH, pol >, rib >$
$t.StrK$	$< g.StrK,\ pol >$	$< g.StrK, pol >$
$StrK$	$< t.StrK,\ rib >$	$<< g.StrK, pol >, rib >$
$(a.34)$	$< (34),\ StrH >$	$< (34), << g.StrH, pol >, rib >>$
(35)	$< (a.34),\ (28) >$	$<< (34), << g.StrH, pol >, rib >>, (28) >$
(41)	$< (35),\ (40) >$	$<<< (34), << g.StrH, pol >, rib >>, (28) >, (40) >$
(42)	$< (41),\ DeH >$	$<<<< (34), << g.StrH, pol >, rib >>, (28) >, (40) >, << g.DeH, pol >, rib >>$
STR	$< (42),\ StrK >$	$<<<<< (34), << g.StrH, pol >, rib >>, (28) >, (40) >, << g.DeH, pol >, rib >>, << g.StrK, pol >, rib >>$

Notes: 'Name' - Name of each one of the elements in the set of internal elements, $\mathbf{S_i}$, of the SI for in the streptomycin (STR) SI (Table S1-2; see Table S1-3 for the keys of the named elements). 'Condensed' - Condensed formula for the element in 'Name'; binary operator, see also Table 2 in main text. ' Expanded' - Expanded formula found for the corresponding element in 'Name'; the result of the C2E algorithm applied to that structure.

streptomycin (STR), do not include this compound among the components needed for its synthesis, i.e., '*to synthesize streptomycin the cell do not need preexistent molecules of streptomycin*'.

S1-3. Implications of the C2E algorithm

As shown in the main text, the C2E algorithm can give a unequivocal classification of essentiality only for internal elements included into the corresponding $\mathbf{S_i}$ set.

As noted in the main text, essentiality rule **ER1** could classify as essential only elements in $\mathbf{S_i}$, while **ER2** could also include as essential elements in $\mathbf{G}$ or $\mathbf{S_e}$. A first example can be seen in Table S1-1 that present the expanded formulae for the elements of $\mathbf{S_i}$ in the RNA polymerase. All formulae in the 'Expanded' column of this table include within its operands the corresponding element in the 'Name' column; i.e., all the elements in the 'Name' column have a recursive formula and thus by **ER1** all of them are essential. By applying **ER2** we found that also all non internal elements named at the SI, say the ones in $\mathbf{G}$ = { `g.bp, g.b, g.o, g.a` } (the genes for each one of the RNA polymerase subunits), as well as the only external element named in the SI, $\mathbf{S_i}$ = { `rib` }, are classified as essential, because they appear as operands in one or more of the expanded formulae in column 'Expanded'. In contrast, none of the elements that intervene in the synthesis of streptomycin ('`STR`'), which expanded formulae are shown in Table S1-4, is recursive and thus none is classified as essential. Even when the ribosome ('`rib`') –an intrinsically essential element of the cell, appears as operand in various of the expanded formulae of Table S1-4, its essentiality is not evident given that none of the internal structures of this SI is classified as essential and thus there is no information to judge its essentiality (by either rule **ER1** or **ER2**). This shows the dependence of our essentiality rules on the quantity of information given by the SI analyzed. In conclusion, elements that are not classified as essential by the information in an SI could be in fact essential for cell functioning –but the SI is not giving enough information for such classification. But in contrast, if an element is classified as essential, such conclusion is definite; no further information added to the SI could change such judgment.

With reference to the essentiality rule **ER3**, we could give a mathematical representation for this rule by defining the genome replication 'operation', say $\mathfrak{D}(\mathbf{G}) \Rightarrow 2\mathbf{G}$, implying with this symbolic representation that the complete genome, '**G**' will be duplicated. Furthermore, the synthesis of all the elements needed to perform this operation in a given cell can be written as a list of binary operators; an SI, say SI^{**}, containing the synthesis definition for all elements necessary for genome replication. Then, **ER3** can be trivially rewritten as

Essentiality rule 3' (ER3'): Essentiality of genome replication machinery.:
s^* is an essential element for genome replication if the absence of s^* impedes the $\mathfrak{D}(\mathbf{G}) \Rightarrow 2\mathbf{G}$ operation.

nevertheless, this rule does not give any algorithmic guideline to discover the elements of such group from a computational manipulation –as did rules **ER1** and **ER2**, thus, we must employ experimental methods to search for such elements, as done, by different approaches and in different organisms, as done for example in (Gerdes et al., 2003; Kobayashi et al., 2003; Berquist et al., 2007; Blomen et al., 2015; Wang et al., 2015).

S1-4. Notes and prerequisites

The remaining part of this document corresponds to computational examples for "On an algorithmic definition for the components of the minimal cell", and can be read in two ways, say, concentrating in the concepts and results presented, disregarding computational details –presented as examples with the R[1] (R Core Team, 2016) package 'InterPlay', or additionally following the step by step R examples.

In the latter case you must install 'InterPlay' using the supplementary file '**S1 Binary**' included in the manuscript "*On an algorithmic definition for the components of the minimal cell*" (the file corresponding to '**S1 Binary**' was named `S1Binary.tar.gz`, and for installation that file must be present in your R working directory). To install the package you could use the command '`R CMD INSTALL S1Binary.tar.gz`' in your operative system command window, or follow instructions at Install Packages from Repositories or Local Files. If you have any problem with the installation please send an e-mail to Octavio Martinez, including the word 'InterPlay' in the subject.

You will also need to install the R package 'igraph' (Csardi and Nepusz, 2006) and include in your R environment the code in the supplementary file '**S2 Text**' (which was uploaded to PLOS ONE as `S2Text.txt`). To do this you must copy that file into your R working directory and type '`source("S2Text.txt")`' at the R prompt ('`>`').

R commands and output are given here in numbered boxes contained within remarks. In these boxes (see `BOX 1` below) the R prompt, '`>`', is included, but, if reproducing the commands in R this prompt must be excluded; you can copy and paste the command to obtain the output which is shown within the box. We assume that you are familiar with R basic use. If you want to follow and reproduce the R commands given in the numbered `BOX`es, it is important that you do so in the same order than such boxes are presented (`BOX 1`, `BOX 2`, $\cdots$, etc.), because in some cases results of a box will depend on operations performed in a previous one.

In case of comments or troubles about our package, please send an e-mail to Octavio Martinez, including the word 'InterPlay' in the subject.

```
######################################## BOX 1 #######################################

# Loading 'InterPlay' (you must have the InterPlay package installed!)
> library(InterPlay)
> ? InterPlay # Start (and browse) the help for the package.
```

[1] Web links which you can follow are given in blue.

```
# Additionally you may include the functions in file  "S2Text.txt"
# After putting that file into your working directory type
> source("S2Text.txt")

# To visualize graphs produced from SIs you need also
# to install the "igraph" R package, and then type
> library(igraph)

####################################### END BOX 1 #######################################
```

S1-5. Computational representation of synthesis interactomes (SIs)

As discussed in the main text, binary operators of the form '$\langle s_{ia}, s_{ib}\rangle \Rightarrow s_i$' can be used to represent the synthesis of cell element 's_i' from the previously existent elements 's_{ia}' and 's_{ib}', which are the operands of the binary operator. Here a synthesis interactome (SI) is defined as a finite collection of different binary operators which summarizes the synthesis of one or more cell elements as function of other. Table S1-5 (as Table 1 in main text) shows a putative SI with k rows.

Table S1-5. **The synthesis interactome (SI).**

Name	Binary operator
s_1	$\langle s_{1a}, s_{1b}\rangle$
s_2	$\langle s_{2a}, s_{2b}\rangle$
$\cdots$	$\cdots$
s_i	$\langle s_{ia}, s_{ib}\rangle$
$\cdots$	$\cdots$
s_k	$\langle s_{ka}, s_{kb}\rangle$

The conditions for a well formed SI are that i) all the names in column 'Name' must be different and ii) All operators in column 'Binary operator' must also be different (see legend of Table 1 as well as '**Mathematical addendum**' in main text). (i) implies that the synthesis of a given structure, say, s^* is defined in a unique way and (ii) implies that a binary operator always result in the synthesis of the same element. SIs can be represented in R (or other computer environments) as tables with three columns, containing the names of elements (column 'Name' in Table S1-5; elements 's_i'), and two columns for the left and right hand side of each binary operator, say, the 's_{ia}' and 's_{ib}' in Table S1-5, and k rows –one for each $i = 1, 2, \cdots, k$ binary operator. BOX 2 shows examples of tables that do not represent well formed SIs.

```
######################################### BOX 2 #########################################
# Example of a data.frame that does not fulfill (i)
> temp <- data.frame(c("a", "a"), c("b", "c"), c("d", "e"), stringsAsFactors=F)
> names(temp) <- c("name", "o1", "o2")
> temp
  name o1 o2
1    a  b  d
2    a  c  e

# See the help for function "normalize.SI"
> ? normalize.SI
```

```
# Check if "temp" is a valid SI
> normalize.SI(temp)
Error in normalize.SI(temp) :
  Not valid interactome: Repeated names of structures ('name')

# Example of a data.frame that does not fulfill (ii)
> temp <- data.frame(c("a", "f"), c("b", "c"), c("c", "b"), stringsAsFactors=F)
> names(temp) <- c("name", "o1", "o2")
> temp
  name o1 o2
1    a  b  c
2    f  c  b
> normalize.SI(temp)
Error in normalize.SI(temp) :
  Not valid interactome: Rows 1 and 2 have the same binary operator!

# NOTE: this last result is given because even when rows 1 and 2 of temp are
# "different" they represent the same binary operator, because binary operators
# are commutative, i.e., <b, c> = <c, b>.
####################################### END BOX 2 #######################################
```

S1-5.1. **Latent sets in an SI.** In Fig. 1 of the main text we presented a Venn diagram with the main subsets of elements cell. An SI will have information about some of such components, and here we dente as **S** the set of all elements present in a given SI. We can always break down **S** into the disjoint sets **G** (the set of genomic elements), $\mathbf{S_i}$ (the set of internal elements) and $\mathbf{S_e}$ (the set of external elements), where

$$\mathbf{S} = \mathbf{G} \cup \mathbf{S_i} \cup \mathbf{S_e}$$

and

$$\mathbf{G} \cap \mathbf{S_i} = \phi; \mathbf{G} \cap \mathbf{S_e} = \phi; \mathbf{G} \cap \mathbf{S_e} = \phi$$

Any well formed SI will contain some information about the elements that form these sets, as exemplified in BOX 3.

```
######################################### BOX 3 #########################################
> ? dummy1.SI # Help for this small interactome.
> data(dummy1.SI) # Load the dataset
> dummy1.SI # Shows the dataset
  name o1 o2
1    a  b  c
2    b  a  d
3    f  e  h
4    g  f  f
5    i  a  b

# Now we will 'normalize' dummy1.SI and obtain information
# about the sets defined within it
> ? normalize.SI # Help for that function
> normalize.SI(SI = dummy1.SI, give.sets = TRUE)
$SI
  name o1 o2 set.o1 set.o2 type.n type.o1 type.o2 structure
1    a  b  c     Si     Se
2    b  a  d     Si     Se
```

```
3    f  e  h     Se     Se
4    g  f  f     Si     Si
5    i  a  b     Si     Si

$sets
$sets$G
character(0)

$sets$Si
[1] "a" "b" "f" "g" "i"

$sets$Se
[1] "c" "d" "e" "h"
####################################### END BOX 3 #####################################
```

From BOX 3 we can note that ‘`normalize.SI`’, aside checking that the SI is a valid interactome, add columns `set.o1, set.o2, type.n, type.o1, type.o2` and `structure` to the corresponding `data.frame`.

The meaning of these columns and the sets infer from the SI by ‘`normalize.SI`’ are presented in Table S1-6. All but the first three columns (`name, o1` and `o2`) are optional in the initial input of a pre-formatted SI; columns `set.o1, set.o2, type.n, type.o1, type.o2` and `structure` will be added in the output (when they are not already present in the input), and columns `set.o1` and `set.o2` will be automatically filled by ‘`normalize.SI`’ with the rules given in Table S1-6.

For real or realistic SIs the input of the columns could be performed in an spreadsheet program (as Excel or alike) and imported into R. In such case, extra columns as ‘`Comments`’ or other could be added for the researcher reference; however, such columns will be deleted and ignored by the InterPlay functions (for an example see help for dataset ‘`dummy3.SI`’ and BOX 4 below).

```
######################################### BOX 4 #######################################
> ? dummy3.SI # See help for that dataset.
> data(dummy3.SI)
> dummy3.SI # See the dataset (output not shown!)
> normalize.SI(dummy3.SI, give.sets = TRUE) # Output not shown!
> ? data(int.SI) # See help for that dataset.
> data(int.SI) # An integrated SI.
> head(int.SI) # Browse the first 6 lines of the SI (output not shown)
int.SI[int.SI$structure == 'pol',] # See all columns that define strucutre "pol"
#  (output not shown)
####################################### END BOX 4 #####################################
```

S1-5.2. **The ‘condensed to expanded’ (C2E) algorithm.** Apart from fulfilling conditions (i) and (ii), ‘interesting’ SIs must contain compressed information about the synthesis of elements, that could be extracted by a chain of substitutions on the binary operators. A valid substitution on binary operators is defined by

- Assume that a row of an SI contains ‘Name’ = a and ‘Binary operator’ = $< b, c >$.
- If there exist other SI row that contains ‘Name’ = b and ‘Binary operator’ = $< d, e >$ then a ‘valid substitution’ for a in the formula $< b, c >$ is given by $<< d, e >, c >$ and
- If there exist other SI row that contains ‘Name’ = c and ‘Binary operator’ = $< f, g >$ then a ‘valid substitution’ for a in the formula $< b, c >$ is given by $< b, < f, g >>$.

TABLE S1-6. Contents in a normalized SI.

First component (named 'SI' or unique component when '`give.sets = FALSE`')	
Column	Content
`name`	A character vector with the names of elements defined in the SI (the set of internal elements $\mathbf{S_i}$)
`o1`	A character vector with the left hand side operand of the binary operator for element in column `name`.
`o2`	A character vector with the right hand side operand of the binary operator for element in column `name`.
`set.o1`	A character vector with the name of the set to which `o1` belongs.
`set.o2`	A character vector with the name of the set to which `o2` belongs.
`type.n`	A character vector with the type of component that the element on column `name` has.
`type.o1`	A character vector with the type of component that the element on column `o1` has.
`type.o2`	A character vector with the type of component that the element on column `o2` has.
`structure`	A character vector with the name of the structure defined in the SI.

Second component (a list with 3 `sets`)	
Component	Content
`G`	A character vector with the names in the set **G** (when the name of the element begins with '`g.`').
`Si`	A character vector with the names in the set $\mathbf{S_i}$ (these are the same elements that appear in the column '`name`').
`Se`	Character vector with the names in the set $\mathbf{S_e}$ (elements in columns '`o1`' or '`o2`' which are not in **G** or $\mathbf{S_e}$).

Note that applying the definitions we can obtain an 'expanded' formula for a given by $<< d, e >, < f, g >> \Rightarrow a$, etc. (see also '**Mathematical addendum**'). In fact, the C2E algorithm implements nested substitutions to 'expand' formulae for all the elements in the column 'Name' of a given SI. An example is presented in BOX 5.

```
########################################## BOX 5 ########################################
> data(dummy1.SI) # Load the dataset
> dummy1.SI # Shows (again) the dataset
  name o1 o2
1    a  b  c
2    b  a  d
3    f  e  h
4    g  f  f
5    i  a  b
> ? C2E # Help for function implementing the "Condensed to Expanded algorithm".
> C2E(s="a", SI=dummy1.SI) # Expands the formula '<b, c>'
[1] "<<a,d>,c>"
> C2E(s="a", SI=dummy1.SI) # Expands the formula '<b, c>'
[1] "<<a,d>,c>"
> ? expandAll # A wraper for C2E to try to expand all elements.
> expandAll(dummy1.SI)
  name condensed      expanded
1    a     <b,c>     <<a,d>,c>
2    b     <a,d>     <<b,c>,d>
3    f     <e,h>         <e,h>
4    g     <f,f> <<e,h>,<e,h>>
```

```
5    i     <a,b>     UNDECIDED

# What happened with the expansion of the formula for 'i'?
# Try to expand the formula for 'i' ('<a,b>')

# a) Print steps and use a maximum of 5 cycles of substitution
C2E(s="i", SI=dummy1.SI, print.steps = TRUE, max.cycles = 5)
[1] "1 - i"
[1] "2 - <a,b>"
[[1]]
[1] "UNDECIDED"

[[2]]
[1] "<<<<<<<a,d>,c>,d>,c>,d>,c>,<<<<<b,c>,d>,c>,d>,c>,d>>"

# It is evident that C2E fell into an infinite loop;
# see that by increasing the max.cycles
> C2E(s="i", SI=dummy1.SI, max.cycles = 10)
[[1]]
[1] "UNDECIDED"

[[2]]
[1] "<<<<<<<<<<<b,c>,d>,c>,d>,c>,d>,c>,d>,c>,d>,c>,<<<<<<<<<<<a,d>,c>,d>,c>,d>,c>,d>
      ,c>,d>,c>,d>>"
####################################### END BOX 5 #######################################
```

From BOX 5 we can easily 'guess' how the C2E algorithm works. In fact, given an initial formula for an element in the form of a binary operator, C2E greedily makes all possible substitutions in the initial formula, by iteratively reviewing the rows of the SI. This is explained in detail and illustrated by pseudo-code in the following paragraphs.

Expressions for binary operators in Table S1-5, that here we denote as '$\mathcal{C}(s_i)$' (column 'Binary operator' in Table S1-5), are 'condensed', in the sense of having only two operands, s_{ia} and s_{ib}. Assume that by inspecting Table S1-5 we find that the formula for s_{ia} (only if $s_{ia} \in \mathbf{S_i}$) is equal to $\mathcal{C}(s_{ia}) = \langle s'_{ia}, s'_{ib}\rangle$. Then we can substitute the value of $\mathcal{C}(s_{ia})$ in the original expression for $\mathcal{C}(s_i)$ to obtain a new expression for $\mathcal{C}(s_i)$, say $\mathcal{C}_1(s_i) = \langle\langle s'_{ia}, s'_{ib}\rangle, s_{ib}\rangle$, where the subindex 1 in $\mathcal{C}$ denotes that we have carried out one substitution to obtain such formula. We could repeat the described substitution process to obtain more 'expanded' formulae, until we exhaust the possibilities for substitution. By iterating the substitution process we will always obtain 'well formed strings', which can be converted into the original condensed formula. To formalize the substitution process we define as $E(X)$ the set of operands that exist into a well formed formula. For example, assume that at some point in the substitution process we find an expression X given by

$$X = \langle\langle s_3, s_4\rangle, \langle s_5, \langle\langle s_3, s_4\rangle, s_2\rangle\rangle\rangle$$

then the set $E(X)$ will be given by all the distinct operands found in expression X, that is

$$E(X) = \{s_2, s_3, s_4, s_5\}$$

Also, we define the operation '$S(X)$' as the substitution in X of all elements of $\mathbf{S_i}$ that exist in the string X by the corresponding binary operators. Thus for example, having X as above, and assuming that we find that only $s_2 \in \mathbf{S_i}$ and that the formula for s_2 in the interactome is '$\langle s_x, s_z\rangle$' then we obtain

$$S(X) = \langle\langle s_3, s_4\rangle, \langle s_5, \langle\langle s_3, s_4\rangle, \langle s_x, s_z\rangle\rangle\rangle\rangle$$

With this notation we can define an algorithm to find an 'expanded' formula from a 'condensed' one, say the C2E algorithm (from 'condensed' to 'expanded'). C2E is presented in the following pseudo-code, implemented into 'InterPlay' as function '`C2E`'.

The C2E ($\mathcal{C}(s) \Rrightarrow \mathcal{E}(s)$) algorithm

1) - INPUT 's' the element name ($s \in \mathbf{S_i}$), 'In', the interactome, and '$m.c$', the maximum of cycles ('loops') of nested substitutions allowed.

2) - SET $f = \mathcal{C}(s)$, $c.c = 0$ and $E = E(f)$.

3) - IF $s \in E$ THEN RETURN f as $\mathcal{E}(s)$.

4) - SET $nf = S(f)$.

5) - WHILE $nf \neq f$ DO

{

5.1) - SET $c.c = c.c + 1$ and $E = E(nf)$

5.2) - IF $c.c > m.c$ RETURN LIST('UNDECIDED', nf)

5.3) - IF $s \in E$ RETURN nf as $\mathcal{E}(s)$.

5.4) - SET $f = nf$ and $nf = S(f)$

}

6) - RETURN nf as $\mathcal{E}(s)$.

In the C2E algorithm, steps 3 and 5.3 are set to avoid a primary infinite loop. Such primary infinite loop will happen if the formula defining s, say f or nf, contains s itself within the operands of the formula. If that is the case, i.e., if $s \in E$, then if step 5.3 is not included, then the substitutions will continue *ad infinitum* within the loop defined in step (5) of the C2E algorithm. Note that steps 3 and 5.3 will always give as output a recursive structure.

There is a second possibility for an infinite loop within this algorithm. This secondary infinite loop will happen always that, at any point, the expanded formula for an element has within its operands a recursive structure. In such case the C2E algorithm will keep substituting the recursive element *ad infinitum.* To scape such possibility, only a maximum number of nested substitutions, '$m.c$', is allowed. If that number of loops is reached within the 'WHILE' loop (5), then C2E return at step 5.2 a list with two components: The word 'UNDECIDED' and the current formula for the structure, nf. In the next section we will see how to deal with that undecided formulae.

If the possible substitutions in a formula had been exhausted, then the nf will not change in two successive evaluations of the 'WHILE' loop defined in step (5) and the condition '$nf \neq f$' will be false (i.e., in that case '$nf = f$') and thus the loop is exited and the final nf is output at step (6).

Thus, the possible results of the C2E algorithm are i) An expanded recursive formula, when the output occurs at steps 3 or 5.3; ii) An 'UNDECIDED' formula when the output occurs at 5.2 or, iii) A non recursive expanded formula when the output occurs at 6). In BOX 5 we saw an example (with '`dummy1.SI`') in which the results of applying '`C2E`' for each row of the SI produced the 3 different output; examining the results of '`expandAll(dummy1.SI)`' –which is a wrapper that applies C2E to each one of the structures of the SI, we see that results for elements '`a`' and '`b`', i.e, the formulae '`<<a,d>,c>`' and '`<<b,c>,d>`' are recursive because they contain the elements being defined –'`a`' and '`b`' respectively. Thus these two formulae were output at step (5.3) and are of type (i), while when making substitutions for the

structure 'i' C2E falls into an infinite loop and outputs 'UNDECIDED' when the maximum number of loops is reached (type (ii) output at (5.2)). In contrast, output for elements 'f' and 'g' –formulae '<e,h>' and '<<e,h>,<e,h>>', are output of type (iii) that happens at step (6) when the formula does not change in two successive loops.

S1-5.3. **Avoiding infinite loops in the search for expanded formulae.** In the previous section we saw that, for an initial condensed formula, the C2E algorithm performs as many substitutions as possible, but falls into an infinite loop when a recursive element is present within a formula that is being expanded. To avoid the possibility of 'UNDECIDED' formula, it is necessary to detect recursive elements 'on the fly' and avoid it subsequent substitution in the expanding formulae. To do this it is necessary to examine the complete SI as a whole, and not one element at the time, as C2E does.

To solve this problem one of us (M.H R-V) designed the "turtle"[2] algorithm, which performs a double cycle of operand substitutions, but as soon as an operand is found within the formula for the corresponding element, such element is "frozen" and it is not substituted in any of the following cycles, avoiding the entrance into infinite loops. A list of all operands is output, including a list of the cases where an operand (in column 'Name' of the SI) was frozen –signaling the fact that its corresponding formula is recursive. In a parallel way, the algorithm keeps track of the formula being expanded and, at the end, it output those expanded formulae. In BOX 6 we present the results of the "turtle" algorithm, as implemented in the function 'findEssential'; see also '**Mathematical addendum**' for a more detailed explanation. An example is presented in BOX 5 with the SI 'dummy1.SI'.

```
######################################### BOX 6 ########################################
# Comparing the results of the C2E algorithm
# (implemente in expandAll) with the results
# of the turtle algorithm (implemente in findEssential).
# As before:
> data(dummy1.SI)
> expandAll(dummy1.SI)
  name condensed      expanded
1    a     <b,c>     <<a,d>,c>
2    b     <a,d>     <<b,c>,d>
3    f     <e,h>         <e,h>
4    g     <f,f> <<e,h>,<e,h>>
5    i     <a,b>     UNDECIDED
# Compare with
> findEssential(dummy1.SI)
  name Recursive Essential o1 o2 Operands          Expanded
1    a      TRUE      TRUE  b  c    a;d;c         <<a,d>,c>
2    b      TRUE      TRUE  a  d    b;c;d         <<b,c>,d>
3    f     FALSE     FALSE  e  h      e;h             <e,h>
4    g     FALSE     FALSE  f  f      e;h     <<e,h>,<e,h>>
5    i     FALSE     FALSE  a  b  b;c;a;d <<<a,d>,c>,<a,d>>
####################################### END BOX 6 ######################################
```

In BOX 6 we can see that 'findEssential(dummy1.SI)' gives an explicit expanded formula, equal to '<<<a,d>,c>,<a,d>>', for the structure 'i' (row 5 on the last table at BOX 6). In contrast with C2E (implemented within 'expandAll'), the turtle algorithm was able to give this explicit formula for the synthesis of the 'i' component because the component 'a' was "frozen" as soon as it was recognized that

[2]M. Humberto Reyes-Valdés gave this name to this algorithm by the fact the first implementation was slow; however, we greatly improved the time performance in the current implementation of the 'findEssential' function.

its formula was recursive, and thus avoided infinite loops. Note that components '`a`' and '`b`' in column '`Recursive`' had value '`TRUE`', pointing to the fact that their corresponding formulae are recursive.

A fundamental difference between the C2E and the turtle algorithms is that C2E gives only expanded formulae for the elements in the set $\mathbf{S_i}$ (and only when such formulae are not undetermined by the algorithm), while turtle gives, apart from final expanded formulae, the complete list of operands which entered –at any point, in the obtention of the expanded formulae.

S1-6. Distinguishing essential cell elements in SIs

We have seen that the turtle algorithm within '`findEssential`' marks as recursive elements in which its expanded formula includes, as operand, the element being defined. This implements the first essentiality rule, '**ER1**' (see main text), which can be stated as '*Elements with recursive expanded formula are essential*'. Even when it has not been mentioned here, '`findEssential`' also implement the second essentiality rule, say, **ER2** (see main text), i.e., '*All operands that appear in the expansion of the formula for an essential element are also essential elements*'. Table S1-7 explain the contents of the output given by the '`findEssential()`' function, while BOX 7 demonstrate the implementation of the second essentiality rule in another example.

Table S1-7. Contents in the output of function '`findEssential()`'.

First component (named '`main`' or unique component when '`give.sets = FALSE`')	
Column	Content
`name`	Character: names of elements defined in the SI (the set of internal elements $\mathbf{S_i}$)
`Recursive`	Logical; is the corresponding expanded formula recursive (and thus the corresponding element is essential by rule 1)?
`Essential`	Logical; is the corresponding element essential? (by essentiality rules 1 or 2)
`o1`	A character vector with the left hand side operand of the binary operator for element in column `name`.
`o2`	A character vector with the right hand side operand of the binary operator for element in column `name`.
`Operands`	Character strings with the operands that appeared during formula expansion
`Expanded`	Character strings with the final expanded formulae for the corresponding elements

Second component (a list with 8 `sets`)	
Component	Content
`G`	A character vector with the names in the set $\mathbf{G}$ (when the name of the element begins with '`g.`').
`EG`	Character vector with the names of essential genomic elements (a proper subset of $\mathbf{G}$).
`Si`	A character vector with the names in the set $\mathbf{S_i}$ (these are the same elements that appear in the column '`name`').
`ESi`	Character vector with the names of the essential elements of $\mathbf{S_i}$
`Se`	Character vector with the names in the set $\mathbf{S_e}$ (elements in columns '`o1`' or '`o2`' which are not in $\mathbf{G}$ or $\mathbf{S_e}$).
`ESe`	Character vector with the names of the essential elements in the set $\mathbf{S_e}$.
`E`	Character vector with the names of the essential elements contained in the SI.
`NE`	Character vector with the names of the non essential elements contained in the SI.

```
######################################### BOX 7 ########################################
> data(dummy2.SI) # Load dummy2.SI
> dummy2.SI # Have a look of this SI
  name  o1 o2
```

```
1    a   b  c
2  a.a   a  d
3    d a.a  e
4    e   f  g
> findEssential(dummy2.SI, give.sets = T)
$main
  name Recursive Essential  o1 o2      Operands            Expanded
1    a     FALSE      TRUE   b  c           b;c               <b,c>
2  a.a      TRUE      TRUE   a  d b;c;a.a;e;f;g <<b,c>,<a.a,<f,g>>>
3    d      TRUE      TRUE a.a  e   a;d;f;g;b;c   <<<b,c>,d>,<f,g>>
4    e     FALSE      TRUE   f  g           f;g               <f,g>

$sets
$sets$G
character(0)

$sets$EG
character(0)

$sets$Si
[1] "a"   "a.a" "d"   "e"

$sets$ESi
[1] "a.a" "d"   "e"   "a"

$sets$Se
[1] "b" "c" "f" "g"

$sets$ESe
[1] "b" "c" "f" "g"

$sets$E
[1] "a.a" "d"   "b"   "c"   "e"   "f"   "g"   "a"

$sets$NE
character(0)
###################################### END BOX 7 ######################################
```

Let's carefully analyze the results of '`findEssential(dummy2.SI, give.sets = T)`' presented in BOX 7. In particular it is important to underline that the column '`Operands`' in the '`findEssential`' output gives a list of all operands that were found during the process of formula expansion, and not only the ones that are present in the final expanded formula in column '`Expanded`'. As example take the row 3 of the '`main`' result:

```
  name Recursive Essential  o1 o2      Operands            Expanded
3    d      TRUE      TRUE a.a  e   a;d;f;g;b;c   <<<b,c>,d>,<f,g>>
```

In this case, for element '`d`', the expanded formula, '`<<<b,c>,d>,<f,g>>`', contain operands `b, c, d, f` and `g` but not the element `a`, which is part of the list presented in the result of column '`Operands`': '`a;d;f;g;b;c`'. This is because, at some point in the expansion of the formula for `d`, the operand `a` appeared, but was posteriorly substituted (by the formula `<b,c>`) and thus it does not form part of the final expanded formula. However, **ER2** is about 'operands' that appear (at some point) in the expansion of

a formula. Given that the expanded formula for `d` is recursive, this element was classified as 'essential' (by **ER1**), but because `a` appears as operand in the expansion of the formula for `d`, `a` is classified as essential by the **ER2**. However, if `d` is essential it follows that `a` is also essential, because to synthesize `d` at some point we need the existence of `a`, as stated by **ER2**. As a final result of '`findEssential(dummy2.SI, give.sets = T)`' we have that all 8 elements named in the SI are essential. `a.a` and `d` are essential because their corresponding expanded formulae are recursive (**ER1**), while the remaining 6 elements (`a, b, c, e, f` and `g`) are essential because they appear as operands for `a.a` or `d` (**ER2**).

We can use R functions to define the different sets present in the '`dummy2.SI`' SI to corroborate the results obtained by '`findEssential(dummy2.SI, give.sets = T)`'; this will be useful in cases with more complex SIs. BOX 8 present such calculations.

```
######################################## BOX 8 ########################################
> data(dummy2.SI)
# Let's obtain the set S of all elements named in dummy2.SI
> S <- sort(union(dummy2.SI$name, union(dummy2.SI$o1, dummy2.SI$o2)))
> S
[1] "a"   "a.a" "b"   "c"   "d"   "e"   "f"   "g"
# Keep the results in a temp object
> temp <- findEssential(dummy2.SI, give.sets = T)
# The set of elements which formulae are recursive
> S.R <- temp$main$name[temp$main$Recursive]
> S.R
[1] "a.a" "d"
# The list of operands for "a.a"
> temp$main$Operands[temp$main$name=="a.a"]
[1] "b;c;a.a;e;f;g"
# Presented as a set (vector)
> strsplit(temp$main$Operands[temp$main$name=="a.a"], split=";")[[1]]
[1] "b"   "c"   "a.a" "e"   "f"   "g"
# All operands for both essential components
# (These are essential by essentiality rule 2, ER2):
> E.O <- sort(union(strsplit(temp$main$Operands[temp$main$name=="a.a"], split=";")[[1]],
        strsplit(temp$main$Operands[temp$main$name=="d"], split=";")[[1]]))
> E.O
[1] "a"   "a.a" "b"   "c"   "d"   "e"   "f"   "g"
# And this set is equal to S:
> setdiff(S, E.O)
character(0)
# And we could see the correspondence with results in the
# collection of sets given by "findEssential":
> names(temp$sets)
[1] "G"   "EG"  "Si"  "ESi" "Se"  "ESe" "E"   "NE"
> setdiff(temp$sets$E, E.O) # Must be the empty set:
character(0)
# Correct.
###################################### END BOX 8 ######################################
```

From Box 8 we see that elements `a.a` and `d` of `dummy2.SI` are classified as essential by the recursiveness of their expanded formula (**ER1**), while all 8 elements present in this SI fulfill the **ER2**, as can be seen by the obtention of the set '`E.O`' in BOX 8.

Boxes 9 and 10 presents the analysis of '`dummy3.SI`', which is an SI obtained from '`dummy2.SI`' by adding elements and detailing the types of elements present.

```
####################################### BOX 9 #######################################
# Compare dummy2.SI with dummy3.SI
> data(dummy2.SI, dummy3.SI)
> dummy2.SI
  name  o1 o2
1    a   b  c
2  a.a   a  d
3    d a.a  e
4    e   f  g
> dummy3.SI
  name  o1  o2 set.o1 set.o2       type.n    type.o1        type.o2  structure
1    a   b   c     Si     Si    Apoenzyme    Peptide        Peptide a.a.System
2  a.a   a   d     Si     Si   Holoenzyme  Apoenzyme       Cofactor a.a.System
3    d a.a   e     Si     Si     Cofactor Holoenzyme   Pre-Cofactor a.a.System
4    e   f   g     Se     Se Pre-Cofactor Metabolite     Metabolite a.a.System
5    x a.a   y     Si     Se   Metabolite Holoenzyme     Metabolite a.a.System
6  t.b g.b pol      G     Se        m-RNA       Gene RNA-polymerase a.a.System
7    b t.b rib     Si     Se      Peptide      m-RNA       Ribosome a.a.System
8  t.c g.c pol      G     Se        m-RNA       Gene RNA-polymerase a.a.System
9    c t.c rib     Si     Se      Peptide      m-RNA       Ribosome a.a.System
                                              Comment
1                           'a' is an inactive enzyme
2                          'a.a' is the active enzyme
3    'd' is a cofactor synthesized from 'e' by 'a.a'
4 'e' is a pre-cofactor synthesized from 'f' and 'g'
5     'x' is a metabolite produced from 'y' by 'a.a'
6        The m-RNA for 'b' is obtained from its gene
7                          Peptide 'b' is synthesized
8        The m-RNA for 'c' is obtained from its gene
9                          Peptide 'c' is synthesized
Normalizes dummy3.SI
> normalize.SI(dummy3.SI)
  name  o1  o2 set.o1 set.o2       type.n    type.o1        type.o2  structure
1    a   b   c     Si     Si    Apoenzyme    Peptide        Peptide a.a.System
2  a.a   a   d     Si     Si   Holoenzyme  Apoenzyme       Cofactor a.a.System
3    d a.a   e     Si     Si     Cofactor Holoenzyme   Pre-Cofactor a.a.System
4    e   f   g     Se     Se Pre-Cofactor Metabolite     Metabolite a.a.System
5    x a.a   y     Si     Se   Metabolite Holoenzyme     Metabolite a.a.System
6  t.b g.b pol      G     Se        m-RNA       Gene RNA-polymerase a.a.System
7    b t.b rib     Si     Se      Peptide      m-RNA       Ribosome a.a.System
8  t.c g.c pol      G     Se        m-RNA       Gene RNA-polymerase a.a.System
9    c t.c rib     Si     Se      Peptide      m-RNA       Ribosome a.a.System
Warning message:
In normalize.SI(dummy3.SI) :
  Argument "SI" has more than 9 columns; extra columns will be deleted in the output!
##################################### END BOX 9 #####################################
```

From BOX 9 we can notice that columns `name`, `o1` and `o2` in rows 1 to 4 are equal in both SIs (`dummy2.SI` and `dummy3.SI`), but `dummy3.SI` includes all columns needed for a normalized SI (see Table S1-6), plus

an extra column named 'Comment'. We can also notice that dummy3.SI include 'biological details' absent from the purely abstract case of dummy2.SI. In fact, we now know that a.a is an holoenzyme[3], i.e., an enzyme that needs a cofactor (d) to be in its active form, and also realize that element a is a heterodimer enzyme formed by two peptides, b and c. Even more, we can see that a.a is needed to obtain its own cofactor, d, from a pre-cofactor, e, and that a.a also catalyze a reaction that gives product x from its interaction with y (row 5 of dummy3.SI). We can also observe that rows 6 to 9 define the synthesis of peptides b and c from their corresponding genes and with the participation of the RNA polymerase (pol) to form the mRNAs (t.b and t.c) and the ribosome (rib) to synthesize the peptides from their corresponding mRNAs. Column 'Comment' could be useful for the reader but it is not a formal part of the SI (see Table S1-6) and thus will be eliminated from it when the SI is processed by any of the functions of InterPlay.

In BOX 10 we can see the results of the analysis performed by 'findEssential' in 'dummy3.SI'.

```
######################################## BOX 10 ######################################
# Performs the findEssential analysis including sets.
> temp <- findEssential(dummy3.SI, give.sets = TRUE)
Warning message:
In normalize.SI(SI, give.sets = TRUE) :
  Argument "SI" has more than 9 columns; extra columns will be deleted in the output!
> names(temp)
[1] "main" "sets"
> temp$main
  name Recursive Essential  o1  o2                          Operands
1    a     FALSE      TRUE   b   c          t.b;rib;t.c;g.b;pol;g.c
2  a.a      TRUE      TRUE   a   d   b;c;a.a;e;g.b;pol;rib;g.c;f;g
3    d      TRUE      TRUE a.a   e a;d;f;g;t.b;rib;t.c;g.b;pol;g.c
4    e     FALSE      TRUE   f   g                               f;g
5    x     FALSE     FALSE a.a   y   a;d;y;t.b;rib;t.c;g.b;pol;g.c
6  t.b     FALSE      TRUE g.b pol                           g.b;pol
7    b     FALSE      TRUE t.b rib                       g.b;pol;rib
8  t.c     FALSE      TRUE g.c pol                           g.c;pol
9    c     FALSE      TRUE t.c rib                       g.c;pol;rib
                         Expanded
1   <<<g.b,pol>,rib>,<<g.c,pol>,rib>>
2 <<<t.b,rib>,<t.c,rib>>,<a.a,<f,g>>>
3                 <<<b,c>,d>,<f,g>>
4                             <f,g>
5                     <<<b,c>,d>,y>
6                         <g.b,pol>
7                   <<g.b,pol>,rib>
8                         <g.c,pol>
9                   <<g.c,pol>,rib>
###################################### END BOX 10 ####################################
```

From BOX 10 we can see that essentiality of the first components defined within both SIs, say a, a.a, d and e is consistently confirmed by the analyses in both SIs (findEssential(dummy2.SI, give.sets = T) in BOX 7 and findEssential(dummy3.SI, give.sets = T) in BOX 10). Now we can see in BOX 11 the sets obtained with findEssential(dummy3.SI, give.sets = T).

```
######################################## BOX 11 ######################################
```

[3]See for example the definition of Cofactor.

```
> temp$sets # Obtained from findEssential in BOX 10
$G
[1] "g.b" "g.c"

$EG
[1] "g.b" "g.c"

$Si
[1] "a"   "a.a" "b"   "c"   "d"   "e"   "t.b" "t.c" "x"

$ESi
[1] "a.a" "d"   "b"   "c"   "e"   "a"   "t.b" "t.c"

$Se
[1] "f"   "g"   "pol" "rib" "y"

$ESe
[1] "pol" "rib" "f"   "g"

$E
 [1] "a.a" "d"   "b"   "c"   "e"   "g.b" "pol" "rib" "g.c" "f"   "g"   "a"   "t.b"
[14] "t.c"

$NE
[1] "x" "y"
####################################### END BOX 11 ####################################
```

From the output in BOX 11 we can first notice that we have two genomic elements, '`g.b`' and '`g.c`' in set '`G`' [4] and we see that those two genes are essential, forming the set '`EG`'. This is a result of the fact that both genes enter (as operands) in the expanded formulas to obtain some of the essential structures, as is corroborated in BOX 12.

```
######################################### BOX 12 ######################################
# Corroborating that genes g.b and g.c are included
# within the operands of some essential structures.
> temp.e <- temp$main$name[temp$main$Essential] # List of essential internal structures
> temp.e
[1] "a"   "a.a" "d"   "e"   "t.b" "b"   "t.c" "c"

## Are "g.b" or "g.c" within the operands of the 8 essential structures?
# Make a list with operands for each essential structure
> temp.o <- strsplit(temp$main$Operands[temp$main$Essential], ";")
> temp.o
[[1]]
[1] "t.b" "rib" "t.c" "g.b" "pol" "g.c"
[[2]]
 [1] "b"   "c"   "a.a" "e"   "g.b" "pol" "rib" "g.c" "f"   "g"
[[3]]
 [1] "a"   "d"   "f"   "g"   "t.b" "rib" "t.c" "g.b" "pol" "g.c"
[[4]]
```

[4] Important: genomic elements are identified in InterPlay functions *only* if their names begin exactly with the symbols '`g.`'; thus this convention for names of genomic elements must be followed when constructing SIs.

```
[1] "f" "g"
[[5]]
[1] "g.b" "pol"
[[6]]
[1] "g.b" "pol" "rib"
[[7]]
[1] "g.c" "pol"
[[8]]
[1] "g.c" "pol" "rib"

# Now for g.b:
for(i in 1:8){
if(is.element("g.b", temp.o[[i]])) print(paste("g.b is element of operands
of the essential structure", temp.e[i]))
}
[1] "g.b is element of operands \n\tof the essential structure a"
[1] "g.b is element of operands \n\tof the essential structure a.a"
[1] "g.b is element of operands \n\tof the essential structure d"
[1] "g.b is element of operands \n\tof the essential structure t.b"
[1] "g.b is element of operands \n\tof the essential structure b"

# and for g.c:
for(i in 1:8){
if(is.element("g.c", temp.o[[i]])) print(paste("g.c is element of operands
of the essential structure", temp.e[i]))
}
[1] "g.c is element of operands of the essential structure a"
[1] "g.c is element of operands of the essential structure a.a"
[1] "g.c is element of operands of the essential structure d"
[1] "g.c is element of operands of the essential structure t.c"
[1] "g.c is element of operands of the essential structure c"
######################################## END BOX 12 #####################################
```

Thus in BOX 12 we corroborated that genes `g.b` and `g.c` are essential because they participate (as operands) in the synthesis of 5 essential structures, and thus fulfill **ER2**.

Going back to the sets obtained in BOX 11 in the result of `findEssential(dummy3.SI, give.sets = T)`, we can see that the only non essential structures (in set `NE`) are `x` and `y`. `x` is an internal element (in set `Si`), which synthesis is defined in the row 5 of `dummy3.SI` which contains the binary operator `<a.a, y>`, thus `y` is an external element in set the set `Se`.

Note that 'essentiality', as given in the output of the function '`findEssential`' depends on the full content of the particular SI analyzed. For example, in the context of `dummy3.SI` the (intrinsically) essential elements RNA polymerase (`pol`) and ribosome (`rib`) are classified as 'essential' (into the set `E`), only by the fact that they participate as operands in the synthesis of essential structures. But it is easy to exemplify cases of SIs where intrinsically essential structures (as `pol` and `rib`) play exactly the same roles, i.e., synthesis of mRNAs from genes and synthesis of polypeptides from mRNAs, respectively, but they are not classified as essential if they do not participate as operands into the synthesis of essential structures. This is illustrated in BOX 13.

```
########################################## BOX 13 ########################################
# A case where pol and rib are classified as non essential
> data(dummy1.SI)
```

```
> dummy1.SI
  name o1 o2
1    a  b  c
2    b  a  d
3    f  e  h
4    g  f  f
5    i  a  b
# Assume that "e" is a peptide.
# Let's add a gene "e" as well as its corresponding transcript.
> t.dummy.SI <- rbind(dummy1.SI, c("t.e", "g.e", "pol"), c("e", "t.e", "rib"),
   stringsAsFactors = FALSE)
> t.dummy.SI
  name  o1  o2
1    a   b   c
2    b   a   d
3    f   e   h
4    g   f   f
5    i   a   b
6  t.e g.e pol
7    e t.e rib
> findEssential(t.dummy.SI, give.sets = TRUE)
$main
  name Recursive Essential  o1  o2          Operands
1    a      TRUE      TRUE   b   c             a;d;c
2    b      TRUE      TRUE   a   d             b;c;d
3    f     FALSE     FALSE   e   h t.e;rib;h;g.e;pol
4    g     FALSE     FALSE   f   f   e;h;g.e;pol;rib
5    i     FALSE     FALSE   a   b           b;c;a;d
6  t.e     FALSE     FALSE g.e pol           g.e;pol
7    e     FALSE     FALSE t.e rib       g.e;pol;rib
                      Expanded
1                     <<a,d>,c>
2                     <<b,c>,d>
3          <<<g.e,pol>,rib>,h>
4 <<<t.e,rib>,h>,<<t.e,rib>,h>>
5            <<<a,d>,c>,<a,d>>
6                     <g.e,pol>
7              <<g.e,pol>,rib>

$sets
$sets$G
[1] "g.e"

$sets$EG
character(0)

$sets$Si
[1] "a"   "b"   "e"   "f"   "g"   "i"   "t.e"

$sets$ESi
[1] "a" "b"
```

```
$sets$Se
[1] "c"   "d"   "h"   "pol" "rib"

$sets$ESe
[1] "d" "c"

$sets$E
[1] "a" "b" "d" "c"

$sets$NE
[1] "g.e" "e"   "f"   "g"   "i"   "t.e" "h"   "pol" "rib"
####################################### END BOX 13 #####################################
```

As shown in BOX 13, when intrinsically essential structures as `pol` and `rib` enter into an SI only as operands for the expansion of formulas of non essential elements –as in this case `e`, then the structures `pol` and `rib` will be classified as 'non essential', i.e., in the output they belong to the set `NE` and also to `Se`, but do not exist into `E`. It is important to underline that this is not an error of the method or the algorithm, but a result of the fact that within the context of a particular SI these structures do not fulfill any of the two essentiality rules.

S1-6.1. **Analysis of a more realistic SI.** In the previous sections we exemplified concepts of SI as well as data and algorithms within the InterPlay package with small fictional SIs (`dummy1.SI`, `dummy2.SI` and `dummy2.SI`). In this section we analyze a more complex an realistic SI, say `int.SI` which contains information for the synthesis of the ribosome (`rib`) from the genes, peptides and ribosomal RNAs which integrate this structure, RNA polymerase (`pol`) from the genes and sub units (peptides) and, finally, for the synthesis of the metabolite streptomycin (`STR`).

BOX 14 shows the first steps to summarize the information present in the SI `int.SI`.

```
######################################### BOX 14 #######################################
> data(int.SI) # Makes the data available
> class(int.SI) # Which class of structure is it?
[1] "data.frame"
> nrow(int.SI) # With how many rows?
[1] 184

# Let's normalize this SI, including its sets:
> int.SI.n <- normalize.SI(int.SI, give.sets = TRUE)
# We know by nrow(int.SI) that there are 184 elements defined within int.SI
# But, what are the numbers of other structures (in G and Se)?
> names(int.SI.n$sets) # To remaind us about the names of the sets
[1] "G"  "Si" "Se"
> length(int.SI.n$sets$G) # Number of genomic elements.
[1] 62
> length(int.SI.n$sets$Si) # Number of internal elements.
[1] 184
> length(int.SI.n$sets$Se) # Number of external elements.
[1] 3
# Let's define the set of all elements named in int.SI
> S.int <- union(int.SI.n$sets$Si, union(int.SI.n$sets$Se, int.SI.n$sets$G))
> length(S.int)
```

```
[1] 249
# Looking at the first 6 rows of int.SI.n$SI
> head(int.SI.n$SI)
     name        o1        o2 set.o1 set.o2   type.n    type.o1         type.o2
1     rib     rs50S     rs30S     Si     Si ribosome    rib.sub         rib.sub
2   rs30S   rRNA16S rp30S1-21     Si     Si  rib.sub    rib.RNA protein complex
3 rRNA16S g.rRNA16S       pol      G     Si     gene       gene          enzyme
4  rp30S1  t.rp30S1       rib     Si     Si  peptide transcript        ribosome
5  rp30S2  t.rp30S2       rib     Si     Si  peptide transcript        ribosome
6  rp30S3  t.rp30S3       rib     Si     Si  peptide transcript        ribosome
  structure
1       rib
2       rib
3       rib
4       rib
5       rib
6       rib

# How many different structures are defined within int.SI.n$SI?
> unique(int.SI.n$SI$structure)
[1] "rib" "pol" "STR"
# How many rows of int.SI.n$SI define each one of these structures?
> table(int.SI.n$SI$structure)

pol rib STR
 12 161  11
####################################### END BOX 14 ####################################
```

From BOX 14 we see that `int.SI` is an SI with 184 rows which contains information for a total of 249 elements, of which 62 ($\approx$ 25%) are genomic elements (in set $\mathbf{G}$), 184 ($\approx$ 74%) are internal elements defined within the SI (in set $\mathbf{S_i}$) and only 3 ($\approx$ 1%) are external elements not defined within the SI (in set $\mathbf{S_e}$). We also see that inside `int.SI` we have information for the synthesis of three 'structures', say `pol` (RNA polymerase) which is determined by 12 rows of `int.SI` representing $\approx$ 7% of the total, `rib` (ribosome) which is determined by 161 rows of `int.SI` representing $\approx$ 88% of the total and `STR` (streptomycin) which is determined by 11 rows of `int.SI` representing $\approx$ 6% of the total number of rows.

We could analyze the full `int.SI` into an SIngle step, but it will be illustrative to analyze first parts of the SI, beginning with the rows that that define the `pol` (RNA polymerase). BOX 15 presents such analysis.

```
######################################### BOX 15 ######################################
# Segregate the part of int.SI which has to do with pol
> pol.SI <- int.SI[int.SI$structure == 'pol',]
> nrow(pol.SI)
[1] 12
> pol.SI.n <- normalize.SI(pol.SI, give.sets = TRUE)
> pol.SI.n$SI[,1:8] # See this interactome (excluding column "structure")
    name   o1  o2 set.o1 set.o2          type.n         type.o1         type.o2
162 bp-b   bp   b     Si     Si protein complex         peptide         peptide
163   2a    a   a     Si     Si protein complex         peptide         peptide
164   ba bp-b  2a     Si     Si protein complex protein complex protein complex
165  pol    o  ba     Si     Si          enzyme         peptide protein complex
```

```
166 t.bp g.bp pol      G     Si      transcript            gene          enzyme
167  t.b  g.b pol      G     Si      transcript            gene          enzyme
168  t.a  g.a pol      G     Si      transcript            gene          enzyme
169  t.o  g.o pol      G     Si      transcript            gene          enzyme
170   bp t.bp rib     Si     Se         peptide      transcript        ribosome
171    b  t.b rib     Si     Se         peptide      transcript        ribosome
172    a  t.a rib     Si     Se         peptide      transcript        ribosome
173    o  t.o rib     Si     Se         peptide      transcript        ribosome
> pol.SI.n$sets # Examine the sets of elements
$G
[1] "g.a"  "g.b"  "g.bp" "g.o"

$Si
 [1] "2a"   "a"    "b"    "ba"   "bp"   "bp-b" "o"    "pol"  "t.a"  "t.b"  "t.bp"
[12] "t.o"

$Se
[1] "rib"

# Use findEssential to perform the analysis
> fe.pol.SI <- findEssential(pol.SI.n, give.sets=T)
> fe.pol.SI$main[,1:5] # See the first 5 columns of main result
   name Recursive Essential   o1  o2
1  bp-b      TRUE      TRUE   bp   b
2    2a      TRUE      TRUE    a   a
3    ba      TRUE      TRUE bp-b  2a
4   pol      TRUE      TRUE    o  ba
5  t.bp      TRUE      TRUE g.bp pol
6   t.b      TRUE      TRUE  g.b pol
7   t.a      TRUE      TRUE  g.a pol
8   t.o      TRUE      TRUE  g.o pol
9    bp      TRUE      TRUE t.bp rib
10    b      TRUE      TRUE  t.b rib
11    a      TRUE      TRUE  t.a rib
12    o      TRUE      TRUE  t.o rib

# Let's see some of the sets associated
> fe.pol.SI$sets$Se # Structures classified as external (Se)
[1] "rib"
> fe.pol.SI$sets$ESe # Essential structures classified as external (ESe)
[1] "rib"
> fe.pol.SI$sets$E # Essential elements
 [1] "bp-b" "2a"   "ba"   "pol"  "t.bp" "t.b"  "t.a"  "t.o"  "bp"   "b"    "a"
[12] "o"    "rib"  "g.bp" "g.b"  "g.o"  "g.a"
> fe.pol.SI$sets$NE # Non Essential elements
character(0)
####################################### END BOX 15 #####################################
```

From BOX 15 we can see that all elements in the '`pol.SI.n`' are classified as essential; even more all the elements in the set $\mathbf{S_i}$ (internal elements in column '`name`' of the SI) have a recursive expanded formula, and thus fulfill **ER1**. We can also see that the only 'external' element (in set $\mathbf{S_e}$) which is named within

the SI is the ribosome (‘`rib`’) which also result to be essential by fulfilling **ER2**; i.e., it enters as operand for all the elements defined in in ‘`name`’, see ‘`fe.pol.SI$main$Operands`’ (data not shown).

In BOX 16 we analyze the subset of the integrate SI ‘`int.SI`’ which is involved with the synthesis of streptomycin (structure ‘`STR`’).

```
######################################## BOX 16 ######################################
# Obtain the streptomycin SI by selecting rows
# with structure == STR
> str.SI <- int.SI[int.SI$structure == 'STR',]
> nrow(str.SI)
[1] 11
> str.SI
      name     o1   o2 set.o1 set.o2     type.n    type.o1    type.o2 structure
174 t.StrH g.StrH  pol      G     Si transcript       gene     enzyme       STR
175   StrH t.StrH  rib     Si     Si    peptide transcript   ribosome       STR
176  t.DeH  g.DeH  pol      G     Si transcript       gene     enzyme       STR
177    DeH  t.DeH  rib     Si     Si     enzyme transcript   ribosome       STR
178 t.StrK g.StrK  pol      G     Si transcript       gene     enzyme       STR
179   StrK t.StrK  rib     Si     Si     enzyme transcript   ribosome       STR
180 (a.34)   (34) StrH     Se     Si metabolite metabolite    peptide       STR
181   (35) (a.34) (28)     Si     Se metabolite metabolite metabolite       STR
182   (41)   (35) (40)     Si     Se metabolite metabolite metabolite       STR
183   (42)   (41)  DeH     Si     Si metabolite metabolite     enzyme       STR
184    STR   (42) StrK     Si     Si metabolite metabolite     enzyme       STR

# Perform the analysis
> fe.str.SI <- findEssential(str.SI, give.sets = T)
# Show the whole results
> fe.str.SI
$main
     name Recursive Essential     o1   o2
1  t.StrH     FALSE     FALSE g.StrH  pol
2    StrH     FALSE     FALSE t.StrH  rib
3   t.DeH     FALSE     FALSE  g.DeH  pol
4     DeH     FALSE     FALSE  t.DeH  rib
5  t.StrK     FALSE     FALSE g.StrK  pol
6    StrK     FALSE     FALSE t.StrK  rib
7  (a.34)     FALSE     FALSE   (34) StrH
8    (35)     FALSE     FALSE (a.34) (28)
9    (41)     FALSE     FALSE   (35) (40)
10   (42)     FALSE     FALSE   (41)  DeH
11    STR     FALSE     FALSE   (42) StrK
                                                                      Operands
1                                                                g.StrH;pol
2                                                            g.StrH;pol;rib
3                                                                 g.DeH;pol
4                                                             g.DeH;pol;rib
5                                                                g.StrK;pol
6                                                            g.StrK;pol;rib
7                                                 (34);t.StrH;rib;g.StrH;pol
8                                              (34);StrH;(28);g.StrH;pol;rib
```

```
9                                (a.34);(28);(40);(34);t.StrH;rib;g.StrH;pol
10                       (35);(40);t.DeH;rib;(34);StrH;(28);g.DeH;pol;g.StrH
11 (41);DeH;t.StrK;rib;(a.34);(28);(40);g.DeH;pol;g.StrK;(34);t.StrH;g.StrH
                                            Expanded
1                                       <g.StrH,pol>
2                                 <<g.StrH,pol>,rib>
3                                        <g.DeH,pol>
4                                  <<g.DeH,pol>,rib>
5                                       <g.StrK,pol>
6                                 <<g.StrK,pol>,rib>
7                          <(34),<<g.StrH,pol>,rib>>
8                         <<(34),<t.StrH,rib>>,(28)>
9                          <<<(34),StrH>,(28)>,(40)>
10      <<<(a.34),(28)>,(40)>,<<g.DeH,pol>,rib>>
11 <<<(35),(40)>,<t.DeH,rib>>,<<g.StrK,pol>,rib>>

$sets
$sets$G
[1] "g.DeH"  "g.StrH" "g.StrK"

$sets$EG
character(0)

$sets$Si
 [1] "(35)"   "(41)"   "(42)"   "(a.34)" "DeH"    "STR"    "StrH"   "StrK"
 [9] "t.DeH"  "t.StrH" "t.StrK"

$sets$ESi
character(0)

$sets$Se
[1] "(28)" "(34)" "(40)" "pol"  "rib"

$sets$ESe
character(0)

$sets$E
character(0)

$sets$NE
 [1] "g.DeH"  "g.StrH" "g.StrK" "(35)"   "(41)"   "(42)"   "(a.34)" "DeH"
 [9] "STR"    "StrH"   "StrK"   "t.DeH"  "t.StrH" "t.StrK" "(28)"   "(34)"
[17] "(40)"   "pol"    "rib"
###################################### END BOX 16 ####################################
```

From the analysis of '`str.SI`' performed in BOX 16 we can see that none of the elements named in this SI are classified as essential. First, none of the expanded formula for the synthesis of elements in the interactome (set $\mathbf{S_i}$, column '`name`') is recursive and as a consequence none of such elements could be classified as essential by rule **ER1**. For this reason **ER2** is not applied to any of the elements named in the SI; i.e., no operands are classified as essential. The set of external elements in the SI includes `"(28)"` `"(34)"` `"(40)"` `"pol"` and `"rib"`, where the first three are metabolites (intermediates in streptomycin

synthesis; see Table 5 in main text for full names of these compounds), and we also have within this set the the intrinsically essential structures "pol" and "rib", which however in the context of the synthesis of streptomycin are classified as 'non essential'. This is not a contradiction because, as remarked before, essentiality is dependent on the context; if none of the elements defined within the SI ('str.SI' in this case) is classified as essential, then neither will be any of the elements in the set of internal elements of the SI.

We are now going to proceed with the analysis of the subset of the integrate SI 'int.SI' which is involved with the synthesis of the ribosome (structure 'rib'). BOX 17 presents this analysis.

```
######################################## BOX 17 ######################################
# Obtain the ribosome SI by selecting rows
# with structure == 'rib'
> rib.SI <- int.SI[int.SI$structure == 'rib',]
# Performs the analysis
> fe.rib.SI <- findEssential(rib.SI, give.sets = T)
# See first row of main result (summarized output!)
> fe.rib.SI$main[1,] # See only the first row of main results
  name Recursive Essential    o1    o2
1  rib      TRUE      TRUE rs50S rs30S

1 rp50S1-31;rRNA23S+5S;rRNA16S;rp30S1-21;rp50S1-29;rp50S30;t.rp50S31;rib;g.rRNA23S;pol;g.rRNA5S;
{ ... lots of operands ...}
t.rp50S1;t.rp50S2

Expanded
1 <<<rp50S1-30,rp50S31>,<<g.rRNA23S,pol>,<g.rRNA5S,pol>>>,<<g.rRNA16S,pol>,<rp30S1-20,rp30S21>>>

# Are all structures defined in rib.SI classified as essential?
> nrow(fe.rib.SI$main) # Number of rows = Structures defined in the SI
[1] 161
# The number of the ones classified as essential
> length(fe.rib.SI$main$name[fe.rib.SI$main$Essential])
[1] 161

#  The number of the ones classified as having a recursive formula
> length(fe.rib.SI$main$name[fe.rib.SI$main$Recursive])
[1] 44
#  The number of the ones not having a recursive formula
> length(fe.rib.SI$main$name[!fe.rib.SI$main$Recursive])
[1] 117
# First 6 names of the ones with a recursive formula
> head(fe.rib.SI$main$name[fe.rib.SI$main$Recursive])
[1] "rib"    "rs30S"  "rp30S3" "rp30S5" "rp30S7" "rp30S9"
# First 6 names of the ones without a recursive formula
> head(fe.rib.SI$main$name[!fe.rib.SI$main$Recursive])
[1] "rRNA16S" "rp30S1"  "rp30S2"  "rp30S4"  "rp30S6"  "rp30S8"

> names(fe.rib.SI$sets) # Reminding the names of the sets obtained
[1] "G"   "EG"  "Si"  "ESi" "Se"  "ESe" "E"   "NE"
# Compare the lengths of essential and non essential sets
> length(fe.rib.SI$sets$G)
```

```
[1] 55
> length(fe.rib.SI$sets$EG)
[1] 55
> length(fe.rib.SI$sets$Si)
[1] 161
> length(fe.rib.SI$sets$ESi)
[1] 161
> length(fe.rib.SI$sets$Se)
[1] 1
> length(fe.rib.SI$sets$ESe)
[1] 1
> fe.rib.SI$sets$ESe
[1] "pol"
> length(fe.rib.SI$sets$E) # Number of essential
[1] 217
> length(fe.rib.SI$sets$NE) # Number of non essential
[1] 0
######################################## END BOX 17 ######################################
```

In BOX 17 we can see that all 161 structures which synthesis is defined within the SI '`rib.SI`' are essential; however only 44 of them ($\approx$ 27%) have a recursive expanded formula, fulfilling **ER1**, while the remaining 117 ($\approx$ 73%) do not have a recursive formulae but fulfill **ER2**, i.e., those elements appear as operands in one more of the cases of essential elements.

It is important to note that '`findEssential`' relies in the existence of the value of '`name`' within the corresponding '`Operands`' to declare the element in '`name`' as an essential component by the application of the **ER1**. By the way in which the '`Expanded`' formula is obtained within '`findEssential`' –which is guided by the turtle algorithm, there could be cases in which the formula shown in '`Expanded`' will not contain among its operands the term '`name`'. This will happen in highly complex SIs (as '`rib.SI`') because the element in '`name`' is not yet found in the growing expanded formula when such '`name`' is frozen. Expanded formula output by '`findEssential`' are not unique, in the sense that the expansion process can be stop at different points, and the formula will be labeled as 'expanded' even when further substitutions could be possible (for example with the C2E algorithm, which in fact exhaust all possible substitutions). We have seen before that expanded formula obtained by '`findEssential`' and '`C2E`' could differ, and this fact is also illustrated in BOX 18.

```
########################################## BOX 18 ########################################
# Comparing expanded formulae obtained by findEssential
# and C2E for 'rib' (following objects obtained in BOX 17)

# The expanded formula found for rib by findEssential
> fe.rib.exp <- fe.rib.SI$main$Expanded[fe.rib.SI$main$name == 'rib']
# The expanded formula found for rib by C2E
> c2e.rib.exp <- C2E("rib", rib.SI)
# See both formulae
[1] "<<<rp50S1-30,rp50S31>,<<g.rRNA23S,pol>,<g.rRNA5S,pol>>>,<<g.rRNA16S,pol>,
    <rp30S1-20,rp30S21>>>"
[1] "<<<<rp50S1-29,rp50S30>,<t.rp50S31,rib>>,<<g.rRNA23S,pol>,<g.rRNA5S,pol>>>,
    <<g.rRNA16S,pol>,<<rp30S1-19,rp30S20>,<t.rp30S21,rib>>>>"
## Obtains the the set of operands that exist in each formulae
# First for the one obtained with findEssential
> op.fe.rib.exp <- gsub("<", "", fe.rib.exp) # Delete all "<"s!
```

```
> op.fe.rib.exp <- gsub(">", "", op.fe.rib.exp) # Delete all ">"s!
# Finally get the operands as a vector
> op.fe.rib.exp <- sort(unique(strsplit(op.fe.rib.exp, split=",", fixed=TRUE)[[1]]))

# Second for the one obtained with C2E (c2e.rib.exp)
> op.c2e.rib.exp <- gsub("<", "", c2e.rib.exp) # Delete all "<"s!
> op.c2e.rib.exp <- gsub(">", "", op.c2e.rib.exp) # Delete all ">"s!
> op.c2e.rib.exp <- sort(unique(strsplit(op.c2e.rib.exp, split=",", fixed=TRUE)[[1]]))

# Compare...
> op.fe.rib.exp; op.c2e.rib.exp
[1] "g.rRNA16S" "g.rRNA23S" "g.rRNA5S"  "pol"       "rp30S1-20" "rp30S21"
[7] "rp50S1-30" "rp50S31"
 [1] "g.rRNA16S" "g.rRNA23S" "g.rRNA5S"  "pol"       "rib"       "rp30S1-19"
 [7] "rp30S20"   "rp50S1-29" "rp50S30"   "t.rp30S21" "t.rp50S31"

# See the differences
> union(op.fe.rib.exp, op.c2e.rib.exp)
 [1] "g.rRNA16S" "g.rRNA23S" "g.rRNA5S"  "pol"       "rp30S1-20" "rp30S21"
 [7] "rp50S1-30" "rp50S31"   "rib"       "rp30S1-19" "rp30S20"   "rp50S1-29"
[13] "rp50S30"   "t.rp30S21" "t.rp50S31"
# (we have 15 operands in the union of both sets)
> intersect(op.fe.rib.exp, op.c2e.rib.exp)
[1] "g.rRNA16S" "g.rRNA23S" "g.rRNA5S"  "pol"
# (but only 4 in the intersection!)
# Which operands are in the C2E but not in the findEssential formula?
> setdiff(op.c2e.rib.exp, op.fe.rib.exp)
[1] "rib"       "rp30S1-19" "rp30S20"   "rp50S1-29" "rp50S30"   "t.rp30S21"
[7] "t.rp50S31"
# And the oposite
> setdiff(op.fe.rib.exp, op.c2e.rib.exp)
[1] "rp30S1-20" "rp30S21"   "rp50S1-30" "rp50S31"

## Let's isolate the operands found by findEssential for rib
> op.fe.rib <- sort(strsplit(fe.rib.SI$main$Operands[fe.rib.SI$main$name == 'rib'],
   split=";")[[1]])
> length(op.fe.rib) # How many operands for "rib"?
[1] 217
> is.element("rib", op.fe.rib) # Is "rib" recursive by essentiality rule 1 (ER1)?
[1] TRUE

# Are all operands in the expanded formulae contained in the set
# of Operands found with "findEssential"?
> setdiff(op.fe.rib.exp, op.fe.rib) # Must be empty set
character(0)
> setdiff(op.c2e.rib.exp, op.fe.rib) # Must also be empty set
character(0)
####################################### END BOX 18 #####################################
```

In BOX 18 we can see that the expanded formulae for '`rib`' obtained with '`findEssential`' and '`C2E`' differ; in fact, the one obtained with C2E is longer (has more operands), and, importantly, it contains within

its operands the term '`rib`' and thus it is explicitly recursive, while the one obtained by '`findEssential`' does not. However, it is possible (but tedious!) to corroborate that the two formulae are expansions of the original binary operator present in the SI for '`rib`' (`<rs50S, rs30S>`), which were stop at different points. However, the long (217 elements) list of operands (in column '`Operands`' for row in which `name == 'rib'`) does contain the term '`rib`' and thus this structure was correctly classified as essential using **ER1**.

To finish this section BOX 19 presents the results of analyzing the complete SI '`int.SI`'

```
######################################## BOX 19 #######################################
# Load the data
> data(int.SI)
# Which structures are defined within int.SI?
> unique(int.SI$structure)
[1] "rib" "pol" "STR"

# Number of elements defined in int.SI
> nrow(int.SI)
[1] 184

# Perform the analysis (measuring the time that it takes)
> system.time(fe.int.SI <- findEssential(int.SI, give.sets=T))
   user  system elapsed
  8.776   0.078   8.789

## Obtains the lengths of each one of the sets
temp <- fe.int.SI$sets
for(i in 1:length(temp)){
print(paste("Set ", names(temp)[i], "has", length(temp[[i]]), "elements."))
}

[1] "Set  G has 62 elements."
[1] "Set  EG has 59 elements."
[1] "Set  Si has 184 elements."
[1] "Set  ESi has 173 elements."
[1] "Set  Se has 3 elements."
[1] "Set  ESe has 0 elements."
[1] "Set  E has 232 elements."
[1] "Set  NE has 17 elements."

# Which are the genomic elements which are not essential?
> setdiff(fe.int.SI$sets$G, fe.int.SI$sets$EG)
[1] "g.DeH"  "g.StrH" "g.StrK"
# Which are the "external" structures?
> fe.int.SI$sets$Se
[1] "(28)" "(34)" "(40)"
# Which are the non essential structures?
> fe.int.SI$sets$NE
 [1] "g.DeH"  "g.StrH" "g.StrK" "(35)"   "(41)"   "(42)"   "(a.34)" "DeH"
 [9] "STR"    "StrH"   "StrK"   "t.DeH"  "t.StrH" "t.StrK" "(28)"   "(34)"
[17] "(40)"
###################################### END BOX 19 ####################################
```

From BOX 19 we see that the SI '`int.SI`' includes rows defining three structures, `"rib", "pol"` and `"STR"`. We have independently seen the analyses of the rows comprising each one of these these structures previously, but here we analyze the whole SI. After using '`findEssential`' to obtain the results, we printed the number of elements that constitute each one of the groups of elements defined in the SI. We find that the set of all structures named within the SI, **S**, and composed by disjoint sets $\mathbf{G}, \mathbf{S_i}, \mathbf{S_e}; \mathbf{S} = \mathbf{G} \cup \mathbf{S_i} \cup \mathbf{S_e}$ contain 62 ($\approx$ 25%), 184 ($\approx$ 74%) and 3 ($\approx$ 1%) elements, respectively; the total of elements in **S** is $62 + 184 + 3 = 249$. And it turns out that $56/62, \approx 90\%$ of the genomic structures in **G** are essential, $173/184 \approx 94\%$ of the internal structures in $\mathbf{S_i}$ are essential, while all 3 external structures in $\mathbf{S_e}$ are non essential. The three non essential external structures named in `int.SI` are `"(28)", "(34)"` and `"(40)"`, which are metabolites involved in the synthesis of streptomycin (`STR`); the names of such compounds are presented in Table 5 of the main text.

One of the important points of comparing the analysis of the integrated interactome '`int.SI`', contrasting it with the partial analyses of '`pol.SI`', '`str.SI`' and '`rib.SI`' is to see how the integration of information within the whole SI clasifies the roles and essentiality of the structures `"rib"` and `"pol"` –which, as mentioned before are 'intrinsically essential'. In the whole analysis of `int.SI` both structures are 'internal' –because there is synthesis information for both of them, and 'essential' –because the sets of operands of their corresponding formulae include the corresponding structures, fulfilling the **ER1**. In contrast, in the analysis of `pol.SI`, `"rib"` is classified as an external and essential structure; in the analysis of `rib.SI`, `"pol"` is classified as an external and essential structure, while in the analysis of `str.SI`, both `"pol"` and `"rib"` are classified as external and non essential structures –because non of the structures named in `str.SI` is essential. Thus we see that the completeness of information about one structure in part determines the ability of the algorithms to classify them into the 'essential' or 'non essential' categories; if enough information is present within the SI for the synthesis of an structure or component, then the algorithms can categorically and correctly classify them into one of the classes, however, when no synthesis information about the structure is present such classification can vary, depending on the fact that the element in question appears within the operands of an essential element (as `"rib"` in `pol.SI` or `"pol"` in `rib.SI`) or not (as `"rib"` and `"pol"` in `str.SI`).

From the analysis of both, '`int.SI`' and '`str.SI`' we confirm that the secondary metabolite streptomycin (`STR`) is non essential, and neither are any of the primary elements which enter into its synthesis –except for the intrinsically essential structures `"rib"` and `"pol"` for which there is synthesis information in '`int.SI`'. In fact, all elements in the set '`NE`' from the analysis of `int.SI` (see those elements shown in the result of '`fe.int.SI$sets$NE`' in BOX 19) are exclusively involved in the synthesis of streptomycin.

An interesting complementary result of the analysis of SIs is the fact that the number of distinct operands that enter into the synthesis of a given structure gives a relative measure of the complexity of the corresponding structure. Thus, by comparing the number of operands used in the synthesis of a given element (or structure) with the total number of posible operands (the number of elements in the set **S**) we can have an idea of the complexity of such element or structure. BOX 20 presents calculations of elements complexity using the function '`mea.compl`' which is included in the supplementary file '`S2_SupplFunct.txt`' which must be previously loaded for the code in BOX 20 to work.

```
######################################### BOX 20 #######################################
# NOTE:
# Please be sure to have loaded the
# supplementary functions with
# > source("S2Text.txt")

# Also the element fe.int.SI obtained with
# fe.int.SI <- findEssential(int.SI, give.sets=T)
# (see BOX 19)
# must be present in your environment.
```

```
# Let's find the relative complexity i.e.,
# Number of operands used in the synthesis / Number of all possible operands
# for "pol", "STR" and "rib":

> mea.compl("pol", fe.int.SI)
[1] "Element 'pol' has a relative complexity of 32/249 (12.85%)"
> mea.compl("STR", fe.int.SI)
[1] "Element 'STR' has a relative complexity of 166/249 (66.67%)"
> mea.compl("rib", fe.int.SI)
[1] "Element 'rib' has a relative complexity of 232/249 (93.17%)"

# Now, estimate the complexity of each one of the 184
# elements synthesized within int.SI, keeping also the
# structure to which they belong
> comp.int.SI <- data.frame(int.SI$name, int.SI$structure, rep(0, 184), stringsAsFactors=F)
> names(comp.int.SI) <- c("name", "structure", "rel.com")
> head(comp.int.SI)
     name structure rel.com
1     rib       rib       0
2   rs30S       rib       0
3 rRNA16S       rib       0
4  rp30S1       rib       0
5  rp30S2       rib       0
6  rp30S3       rib       0

# Fill the rel.com variable with complexity results
for(i in 1:184){
comp.int.SI$rel.com[i] <- mea.compl(comp.int.SI$name[i], fe.int.SI)
}
# Output not shown!

# See the first rows of the result
> head(comp.int.SI)
     name structure rel.com
1     rib       rib   93.17
2   rs30S       rib   44.58
3 rRNA16S       rib   12.05
4  rp30S1       rib   62.25
5  rp30S2       rib   62.25
6  rp30S3       rib   62.25

# Order the results by decreasing relative complexity
> comp.int.SI <- comp.int.SI[order(comp.int.SI$rel.com, decreasing=T),]
# See the first and last rows of this (reordered) dataset
> head(comp.int.SI)
         name structure rel.com
1         rib       rib   93.17
161 rp50S1-31       rib   80.72
159 rp50S1-29       rib   79.12
157 rp50S1-27       rib   77.91
```

```
155 rp50S1-25       rib   76.71
153 rp50S1-23       rib   75.50
> tail(comp.int.SI)
          name structure rel.com
164         ba       pol   12.05
166       t.bp       pol   12.05
167        t.b       pol   12.05
168        t.a       pol   12.05
169        t.o       pol   12.05
67  rRNA23S+5S       rib   10.04

# Summarize the relative complexity
> summary(comp.int.SI)
     name            structure            rel.com
 Length:184         Length:184         Min.   :10.04
 Class :character   Class :character   1st Qu.:12.45
 Mode  :character   Mode  :character   Median :54.22
                                       Mean   :42.44
                                       3rd Qu.:62.25
                                       Max.   :93.17
> sd(comp.int.SI$rel.com)
[1] 24.84488

# Number of elements which enter into the
# synthesis of each structure:
> table(comp.int.SI$structure)

pol rib STR
 12 161  11
# And in percentage
> round(100*table(comp.int.SI$structure)/184)

pol rib STR
  7  88   6

# Summarize the relative complexity per structure
> summary(comp.int.SI$rel.com[comp.int.SI$structure == "pol"])
   Min. 1st Qu.  Median    Mean 3rd Qu.    Max.
  12.05   12.05   12.25   28.88   62.25   62.25
> summary(comp.int.SI$rel.com[comp.int.SI$structure == "STR"])
   Min. 1st Qu.  Median    Mean 3rd Qu.    Max.
  12.45   13.26   62.65   40.96   63.26   66.67
> summary(comp.int.SI$rel.com[comp.int.SI$structure == "rib"])
   Min. 1st Qu.  Median    Mean 3rd Qu.    Max.
  10.04   12.45   54.22   43.55   62.25   93.17
# Boxplot of rel.com per structure (output not shown):
# > boxplot(rel.com ~ structure, data=comp.int.SI)
####################################### END BOX 20 ####################################
```

From BOX 20 we can see that the relatively less ‘complex’ structure defined within the ‘`int.SI`’ is the RNA polymerase, ‘`pol`’ in which synthesis participate 32 of the 249 operands present in that SI, i.e., it uses

≈ 12.85% of the operands. '`pol`' is followed in relative complexity by '`STR`' with 166/249 (≈ 66.67%) and then by '`rib`' with the highest value of relative complexity, 232/249 (≈ 93.17%). Evidently, this measure of 'relative complexity' can be criticized as misleading, because from the structural and other points of view it is clear, for example, that the RNA polymerase is much more 'complex' than the metabolite streptomycin. However, within the very simplified framework of the SI presented here, and taking into account only the number of operands employed –with tacitly assume that each binary operation as well as every operand has equal complexity or information, the number of operands employed in the synthesis of a given element has some explicative value.

By obtaining the relative complexity of all elements defined in the '`int.SI`' (BOX 20), we find that it ranges between 10 and 93 percent, with a median of 54 an a mean of 42. By measuring the relative complexity of the elements which enter in the synthesis of each structure, we also confirm what was previously observed; the 7 elements that enter into the synthesis of `pol` are in average (28.88) less 'complex' than the 6 that enter into the synthesis of `STR` (40.96), and the average complexity of the 88 elements that enter in the synthesis of`rib` is the highest (43.55).

S1-7. Visualizing SI relations with graph theory

Mathematically a graph is an ordered pair $G = (V, E)$, where V is a set of vertices or nodes and E is a set of edges or lines, which are 2-element subsets of V (Bang-Jensen and Gutin, 2008). In the interactome, cell elements are nodes (elements of the set V), while binding or synthesis define two different types of edges (the E set).

We have seen that algebraic manipulations can determine the essentiality of cell elements defined within an SI. However, visualization of the plots derived from SIs is of great help for a better understanding of the relations existent in SIs, as well as to corroborate the essentiality of SI elements. At least two relations can be visualized as a network from an SI: the 'binding' and the 'synthesis' relations between elements. In both cases the elements of the SI are 'nodes' while the relations are visualized as 'edges' between nodes. 'edges' will be non-directed lines in the case of the 'binding' relation while them will be directed lines or 'arrows' in the second.

S1-7.1. **'binding' and 'synthesis' networks from the RNA polymerase SI.** BOX 21 presents a first example with the networks generated by the RNA polymerase SI.

```
######################################### BOX 21 ########################################
# You musta have installed the "igraph" and "InterPlay" packages
> library(InterPlay); library(igraph) # load the packages
# You must have the file "S2Text.txt" in your working directory
> source("S2Text.txt")
# Load the "int.SI" data and obtain "pol.SI"
> pol.SI <- int.SI[int.SI$structure == "pol",]

# Obtaining the "binding" graph for pol.SI
# See the SI2d function:

> SI2d # (only partial output is shown here!)

# Note the remarks which give some help:
# Produces a graph (in igraph format) from an SI (in InterPlay format)

# Parameters:
# SI - An syntesis interactome (SI)
```

```
# which.one - Which type of graph, "synthesis" or "binding".
# give.sets - Logical, Want the sets ("Si", "G", "Se") in output? FALSE or TRUE.

# Output:

# A list with 2:
# d - A data frame for edges with names "from" and "to".
# nod.at - A data frame with vertices names and attributes

# If give.sets=FALSE a third component of the output
# will contain the sets "Si", "G", "Se".

# Use the function 'SI2d':
> d.binding.pol <- SI2d(SI=pol.SI, which.one="binding", give.sets=T)
# Lets see the components of this object
> names(d.binding.pol) # d.binding.pol is a list!
[1] "d"      "nod.at" "sets"
> head(d.binding.pol$d) # This is the binding graph
  from  to
1   bp   b
2    a   a
3 bp-b  2a
4    o  ba
5 g.bp pol
6  g.b pol
> head(d.binding.pol$nod.at) # This gives "properties" of the nodes
  name set            type structure
1   2a  Si protein complex       pol
2    a  Si         peptide       pol
3    b  Si         peptide       pol
4   ba  Si protein complex       pol
5   bp  Si         peptide       pol
6 bp-b  Si protein complex       pol
# Obtain a graph with the igraph function:
> ? graph_from_data_frame # Ask for help...

# Make a graph (in "igraph" format)
g.pol.bind <- graph_from_data_frame(d.binding.pol$d, directed=F)
> g.pol.bind <- graph_from_data_frame(d.binding.pol$d, directed=F)
> g.pol.bind ## Summarizes this graph
IGRAPH a5feae0 UN-- 17 12 --
+ attr: name (v/c)
+ edges from a5feae0 (vertex names):
 [1] bp   --b    a    --a    bp-b--2a   o    --ba   g.bp--pol g.b --pol g.a --pol
 [8] g.o --pol t.bp--rib t.b --rib t.a --rib t.o --rib

# Plot this graph
# Ignore the remarked functions "tiff" and "dev.off" used to output the plot as a file.
# tiff("plots/polBind.tiff") # NOTE: Shown as "Figure 1".
plot(g.pol.bind, main="Network of binding in \'pol\' SI.", sub="(default parameters)")
# dev.off()
```

```
######################################## END BOX 21 ######################################
```

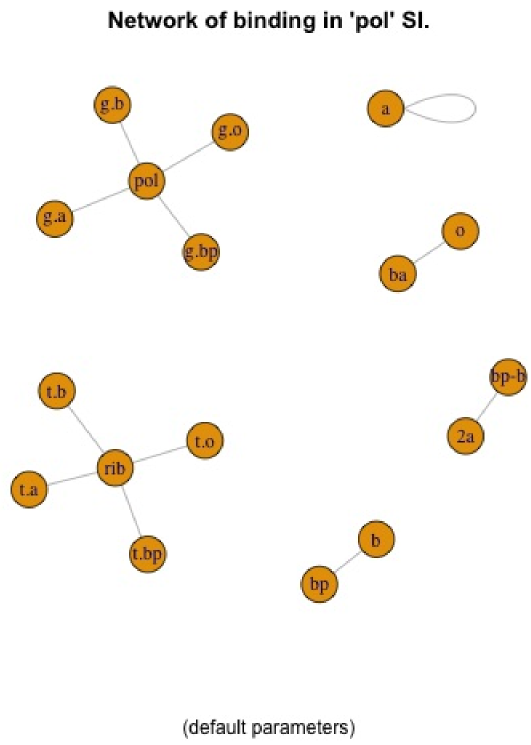

FIGURE 1. Binding between elements in the SI for the RNA polymerase (in '`pol.SI`').

In BOX 21 we see how to use the function '`SI2d`' to extract from an SI either, the binding (shown in the example) or the synthesis relations (to be seen in BOX 22) which exist into an SI. '`SI2d`' gives a list, which first component is a `data.frame` named '`d`', with columns '`from`' and '`to`', indicating that an 'edge' (undirected line) must join the corresponding elements in the row. The second component of the list is another `data.frame` named '`nod.at`' (node attributes), which use will be seen later. The '`d`' part of the list given by '`SI2d`' can be used by the igraph function '`graph_from_data_frame`' to obtain a graph which can be then plotted (see the help of that function which will be repeatedly used in the following examples). For example, the first line printed when the command '`> g.pol.bind`' is given, say `IGRAPH a5feae0 UN-- 17 12 --` ', means that we have an UNdirected (the '`U`' part) graph with '`17`' nodes and '`12`' edges or lines.

By plotting the graph with the command '`plot(g.pol.bind)`' we obtain the result shown in Figure 1. Your results could be different in the positions of the nodes –because there is a random component on that, but the 17 elements of the SI and the 12 binding connections between them will be correctly shown as in Figure 1. By obtaining and ploting the binding network of '`pol.SI`' we are showing explicitly (in Figure 1) the binding operators that exist in such SI. For example, the '`a`' structure has a line connecting '`a`' with itself –because two '`a`' elements exist in the binary operator that determine the structure '`2a`'; see '`pol.SI[2,]`' in your R command window.

We are not going to give more examples of 'binding' graphs that arise from SIs, but will concentrate in the more interesting 'synthesis' graphs latent in SIs. BOX 22 presents the synthesis network determined by '`pol.SI`'.

```
############################################ BOX 22 #######################################
# Obtains the object containing data frames for the synthesis interactome in pol.SI.
> d.pol <- SI2d(pol.SI)
```

```
# Note that we used the defaults of the SI2d function, which are:
# which.one="synthesis" and  give.sets=FALSE

# Let’s see this object
> d.pol
$d
   from   to
1    bp bp-b
2     a   2a
3  bp-b   ba
4     o  pol
5  g.bp t.bp
6   g.b  t.b
7   g.a  t.a
8   g.o  t.o
9  t.bp   bp
10  t.b    b
11  t.a    a
12  t.o    o
13    b bp-b
14   2a   ba
15   ba  pol
16  pol t.bp
17  pol  t.b
18  pol  t.a
19  pol  t.o
20  rib   bp
21  rib    b
22  rib    a
23  rib    o

$nod.at
   name set            type structure
1    2a  Si protein complex       pol
2     a  Si         peptide       pol
3     b  Si         peptide       pol
4    ba  Si protein complex       pol
5    bp  Si         peptide       pol
6  bp-b  Si protein complex       pol
7   g.a   G            gene       pol
8   g.b   G            gene       pol
9  g.bp   G            gene       pol
10  g.o   G            gene       pol
11    o  Si         peptide       pol
12  pol  Si          enzyme       pol
13  rib  Se        ribosome       pol
14  t.a  Si      transcript       pol
15  t.b  Si      transcript       pol
16 t.bp  Si      transcript       pol
17  t.o  Si      transcript       pol
###################################### END BOX 22 ####################################
```

In the cases of graphs obtained for the synthesis of elements, as the case of '`d.pol`', the first component, a data frame with with column names '`from`' and '`to`', determines a directed graph, meaning that instead of simple lines as vertices, we have directed vertices shown as 'arrows'. In the context of an SI, the function '`SI2d`' with the (default) option '`which.one="synthesis"`' will give in the '`d`' component indication of the synthesis path of elements; in that context columns '`from`' and '`to`' mean that the element in column '`from`' is obtained by adding something to the element in column '`to`'. In BOX 23 we will see the plotting of the synthesis graph obtained in '`d.pol`' and in BOX 24 we will see how to decorate this plot with particular attributes of the nodes, present in '`d.pol$nod.at`', which contains columns '`name`', '`set`', '`type`' and '`structure`'.

```
######################################## BOX 23 ######################################
# First let's obtain a graph of the synthesis interactome in d.pol
> g.pol <- graph_from_data_frame(d.pol$d, directed = TRUE)
> g.pol
IGRAPH 67663b8 DN-- 17 23 --
+ attr: name (v/c)
+ edges from 67663b8 (vertex names):
 [1] bp  ->bp-b a   ->2a   bp-b->ba   o   ->pol  g.bp->t.bp g.b ->t.b  g.a ->t.a
 [8] g.o ->t.o  t.bp->bp   t.b ->b    t.a ->a    t.o ->o    b   ->bp-b 2a  ->ba
[15] ba  ->pol  pol ->t.bp pol ->t.b  pol ->t.a  pol ->t.o  rib ->bp   rib ->b
[22] rib ->a    rib ->o
> ? E
> E(g.pol) # Gives the edges sequence in the graph.
+ 23/23 edges from 67663b8 (vertex names):
 [1] bp  ->bp-b a   ->2a   bp-b->ba   o   ->pol  g.bp->t.bp g.b ->t.b  g.a ->t.a
 [8] g.o ->t.o  t.bp->bp   t.b ->b    t.a ->a    t.o ->o    b   ->bp-b 2a  ->ba
[15] ba  ->pol  pol ->t.bp pol ->t.b  pol ->t.a  pol ->t.o  rib ->bp   rib ->b
[22] rib ->a    rib ->o
> ? V
> V(g.pol) # Gives the vertices in the graph.
+ 17/17 vertices, named, from 67663b8:
 [1] bp   a    bp-b o    g.bp g.b  g.a  g.o  t.bp t.b  t.a  t.o  b    2a   ba
[16] pol  rib

# Ignore the remarked functions "tiff" and "dev.off" used to output the plot as a file.
# tiff("plots/polSynth.tiff") # NOTE: Shown as "Figure 2".
plot(g.pol, main="Synthesis network in \'pol\' SI.", sub="(default parameters)")
# dev.off()
###################################### END BOX 23 ####################################
```

In BOX 23 (as well as from BOX 22) we see that the network interactome of the '`pol.SI`' contains 23 relations (edges) between 17 elements (vertices), that are each one of the elements of the **S** set of structures named within the SI '`pol.SI`', and Figure 2 shows the plot of the synthesis network from '`pol.SI`', plotted with the default parameters.

Before proceeding, we can see Figure 3 (presented as Fig. 2 in the main text) to remember that external elements (in set $\mathbf{S_e}$; box (A) in Figure 3) can only have 'departing' or 'out' arrows, as do genomic elements (in set **G**; box (B) in Figure 3), while elements which synthesis is described into the SI, i.e., internal elements in set $\mathbf{S_i}$ (box (C) in Figure 3) have exactly 2 'incoming' or 'in' arrows, because synthesis in an SI is defined by binary operators, and these elements could have any number of departing arrows.

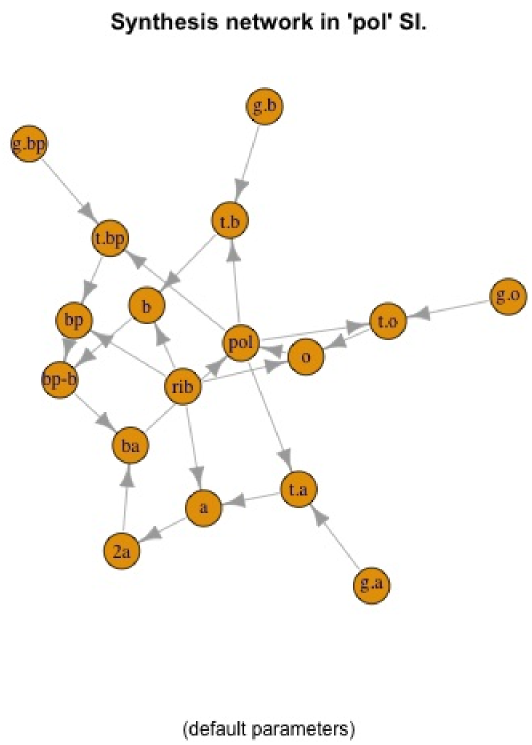

FIGURE 2. Synthesis network in the SI for the RNA polymerase (in 'pol.SI').

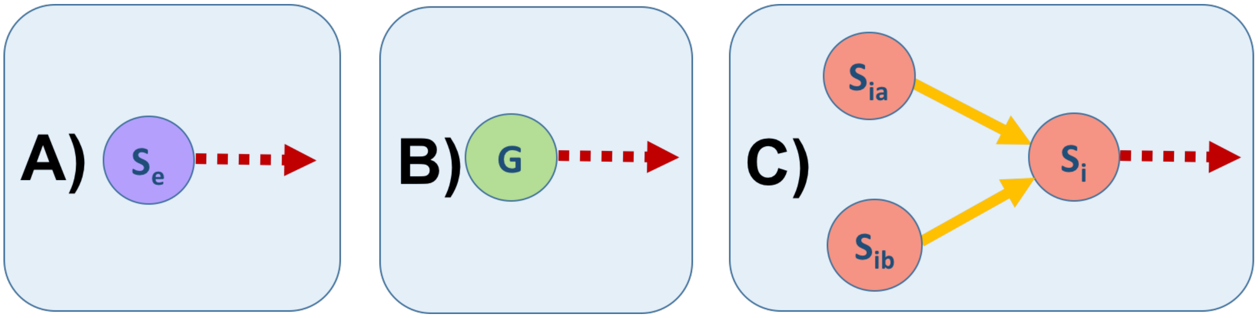

FIGURE 3. Possible cases of edges (arrows) for elements of different sets, $\mathbf{S_e}$, $\mathbf{G}$ and $\mathbf{S_i}$ in A) B) and C) respectively, in a synthesis interactome network.

Figure 2 can be improved, by denoting with different colors, for example, to which set each one of the nodes belong. In BOX 24 we show how to decorate this plot with particular attributes of the nodes, present in the component 'd.pol$nod.at', which contains columns 'name', 'set', 'type' and 'structure'.

```
######################################### BOX 24 #######################################
# We will use the function "map2rain" to select colors for plotting vertices attributes
> map2rain # See the function (not all output shown)
# map2rain
# Utility function to color graphs from an interactome.

# Parameters:
# net - A graph (or "network") as produced by "graph_from_data_frame"
# what - Option for which type of attribute will be color,
```

```
#  possible values are: "type", "set" or "structure"
# alpha - The alpha parameter for the "rainbow" function (>=0, <=1)

# Output:
# A list with two components:
# 1) - col.key (a data frame with "types" and "my.col")
# 2) - A vector with the color definition for each vertice.

# Produces a graph of d.pol which include the attributes of vertices
> g2.pol <- graph_from_data_frame(d=d.pol$d, directed=TRUE, vertices=d.pol$nod.at)
> g2.pol # Note the "attr" (attributes row)
IGRAPH bfccfea DN-B 17 23 --
+ attr: name (v/c), set (v/c), type (v/c), structure (v/c)
+ edges from bfccfea (vertex names):
 [1] bp   ->bp-b a    ->2a   bp-b->ba   o    ->pol  g.bp->t.bp g.b ->t.b  g.a ->t.a
 [8] g.o ->t.o  t.bp->bp   t.b ->b    t.a ->a    t.o ->o    b    ->bp-b 2a  ->ba
[15] ba  ->pol  pol ->t.bp pol ->t.b  pol ->t.a  pol ->t.o  rib ->bp   rib ->b
[22] rib ->a    rib ->o

# Produce an object with colors for g2.pol for attribute "set"
> col.d.pol.set <- map2rain(net=g2.pol, what="set")
# The first component has the color that will be used
# the second the color of each element (vertex)

# Produce a plot with vertices colored by set (not shown)
> plot(g2.pol, vertex.color=col.d.pol.set$colors)

## Improving the plot by modifying  parameters
# For layout:
> set.seed(2018) # (to obtain same results than here)
> temp <- layout_nicely(g2.pol)

# Using that layout:
> old.par <- par() # Keeps parameters default
> par(mai=c(0,0,0,0)) # Modify that (see ? par)

# See the plot with a "nice layout" and colored by "set"
# Adding a legend with the meaning of the set colors
> plot(g2.pol, vertex.color=col.d.pol.set$colors, layout=temp)
> legend("topleft", legend=col.d.pol.set$col.key$types, pch=21,
   pt.bg=col.d.pol.set$col.key$my.col, pt.cex=3.5, cex=1.8, bty="n", ncol=1)
# Previous plot as "Figure 4" in text.

## Now obtain the colors for "type"
 > col.d.pol.type <- map2rain(net=g2.pol, what="type")

# Obtain the plot with elements colored by type
# setting the legend.
plot(g2.pol, vertex.color=col.d.pol.type$colors, layout=temp)
legend("topleft", legend=col.d.pol.type$col.key$types, pch=21,
   pt.bg=col.d.pol.type$col.key$my.col, pt.cex=3.5, cex=1.8, bty="n", ncol=1)
```

```
# Previous plot as "Figure 5" in text.
######################################## END BOX 24 ######################################
```

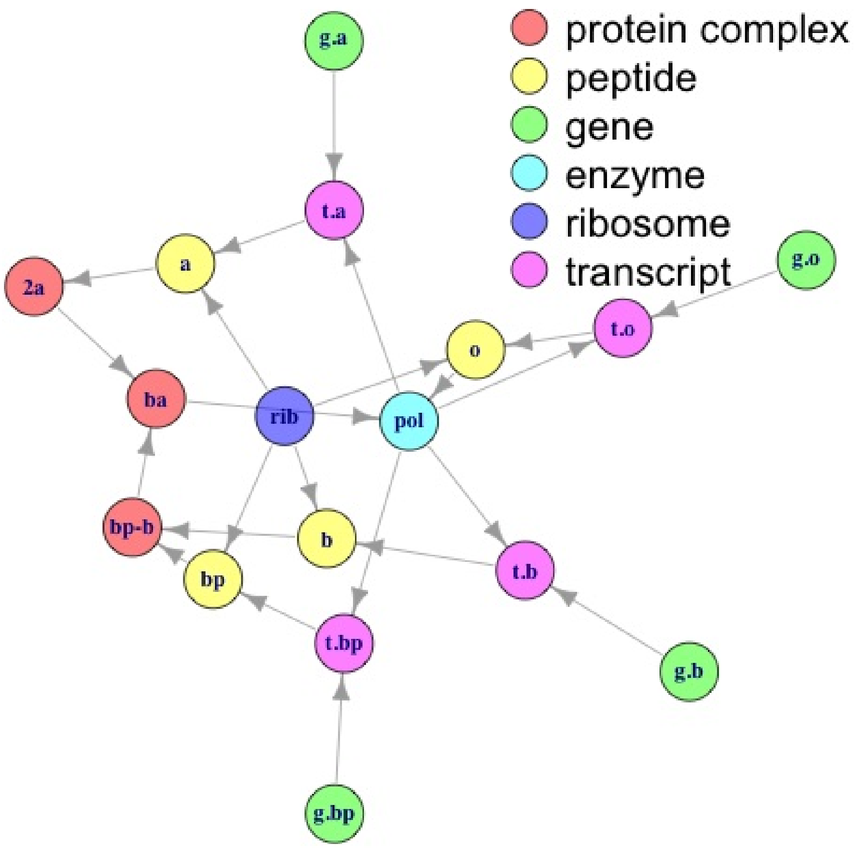

Figure 4. RNA polymerase network with elements colored by set of origin.

In BOX 24 we have seen how to color the synthesis network of the RNA polymerase (from the SI ‘`pol.SI`’) by the set of origin of each one of the elements (Figure 4) or, alternatively by the ‘type’ of component of each one of the elements (Figure 5).

From Figures 2, 4 and 5 can be seen how all the elements of the internal set of ‘`SI2d`’, i.e., the elements for which synthesis information exist in the interactome, `bp-b, 2a, ba, pol, t.bp, t.b, t.a, t.o, bp, b, a` and `o` form part of one or more ‘cycle’ or closed walk. This confirms that all those elements fulfill the **ER1**, because if these elements form a cycle it means that their pre-existence is needed for their synthesis. In these figures we also see that all genomic elements in the interactome, genes `g.a, g.b, g.bp` and `g.o` which code for the corresponding essential peptides `a, b, bp` and `o` are also essential by fulfilling **ER2**, i.e., they are direct operands of essential structures.

Figures 3 to 5 in the main text where produced following the same steps delineated here, i.e., first creating a graph object with our auxiliar function ‘`SI2d`’, for a synthesis network and from this obtaining an igraph object with the function ‘`graph_from_data_frame`’, which then was plotted with elements colored by the colors obtained with our auxiliar function ‘`map2rain`’.

To finish with the presentation of examples, BOX 25 shows the code to obtain the plots of the graph of the synthesis interactome ‘`str.SI`’, which contains information for the synthesis of streptomycin.

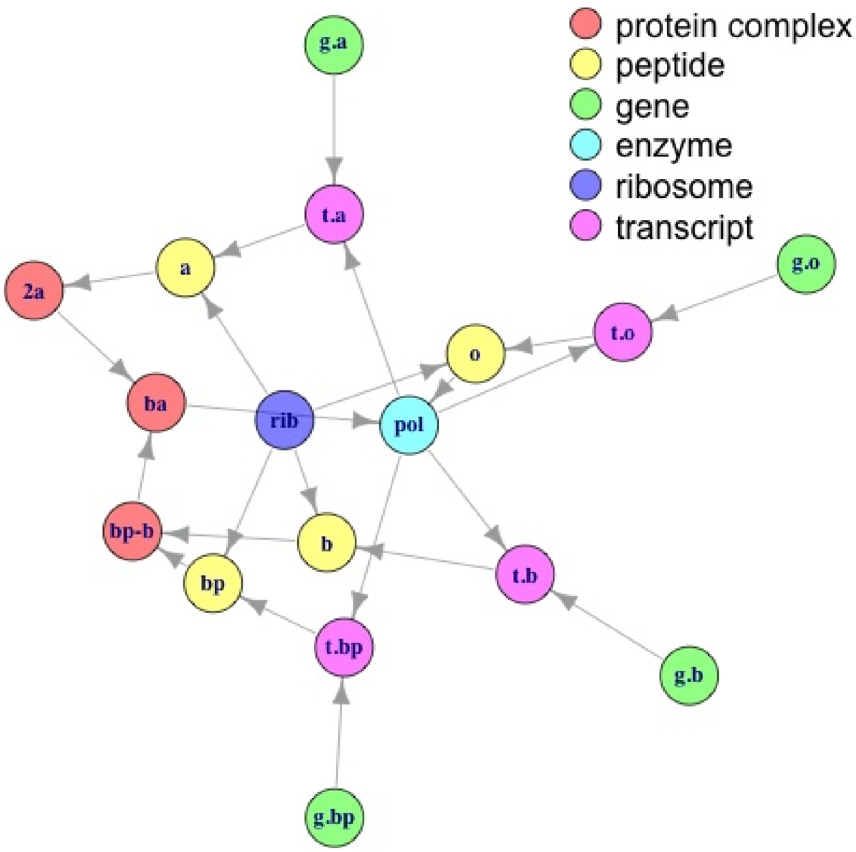

FIGURE 5. RNA polymerase network with elements colored by type of component.

```
######################################## BOX 25 ######################################
# Load the data and obtain str.SI
> data(int.SI)
> str.SI <- int.SI[int.SI$structure == "STR",]

# Obtain an object from int.SI
> d.str <- SI2d(str.SI) # default to obtain "synthesis"

# Obtains a graph from d.str using graph_from_data_frame
> g.str <- graph_from_data_frame(d=d.str$d, directed=TRUE, vertices=d.str$nod.at)

# Set a nice layout for the plot
> set.seed(1959) # (to obtain same results than here)
> temp <- layout_nicely(g.str)

# Obtain colors by set for the plot
> g.str.col.set <- map2rain(net=g.str, what="set")

# Obtain colors by type of element
> g.str.col.type <- map2rain(net=g.str, what="type")

# Obtain the plot of synthesis network colored by set
```

```
# (Output shown in Figure 6)
# tiff("plots/strSet.tiff")
par(mai=c(0,0,0,0))
plot(g.str, vertex.color=g.str.col.set$colors, edge.width=3, vertex.label.cex=1.2,
   vertex.size=23, layout=temp, edge.arrow.size=0.5, vertex.label.font=2)
legend("bottomleft", legend=g.str.col.set$col.key$types, pch=21,
   pt.bg=g.str.col.set$col.key$my.col, pt.cex=3.5, cex=2, bty="n", ncol=1)
# dev.off()

# Obtain the plot of synthesis network colored by type
# (Output shown in Figure 7)
# tiff("plots/strType.tiff")
par(mai=c(0,0,0,0))
plot(g.str, vertex.color=g.str.col.type$colors, edge.width=3, vertex.label.cex=1.2,
   vertex.size=23, layout=temp, edge.arrow.size=0.5, vertex.label.font=2)
legend("topright", legend=g.str.col.type$col.key$types[1:4], pch=21,
   pt.bg=g.str.col.type$col.key$my.col[1:4], pt.cex=3.5, cex=2, bty="n", ncol=1)
legend("bottomleft", legend=g.str.col.type$col.key$types[5:6], pch=21,
   pt.bg=g.str.col.type$col.key$my.col[5:6], pt.cex=3.5, cex=2, bty="n", ncol=1)
# dev.off()
######################################## END BOX 25 ######################################
```

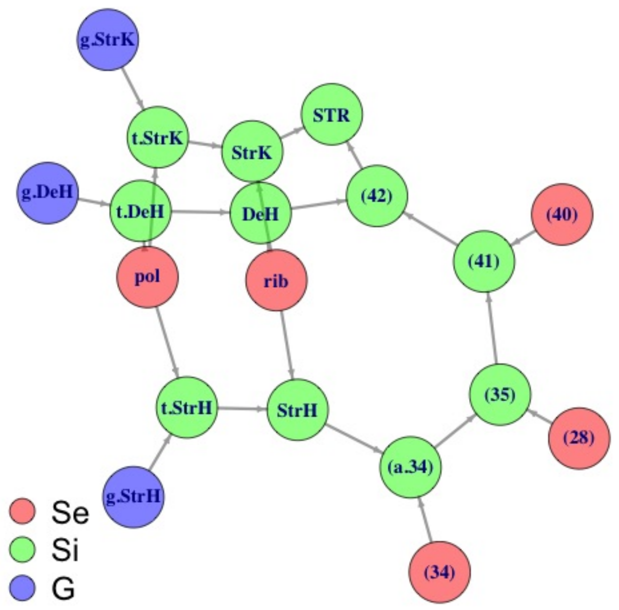

FIGURE 6. Streptomycin synthesis network with elements colored by set of origin (for definitions of names see Table 5 in main text).

From figures 6 and 7 we can see that none of the elements of the synthesis network for a cycle or closed walk, reflecting the facts that within the context of the ‘`str.SI`’ none of its elements can be classified as essential.

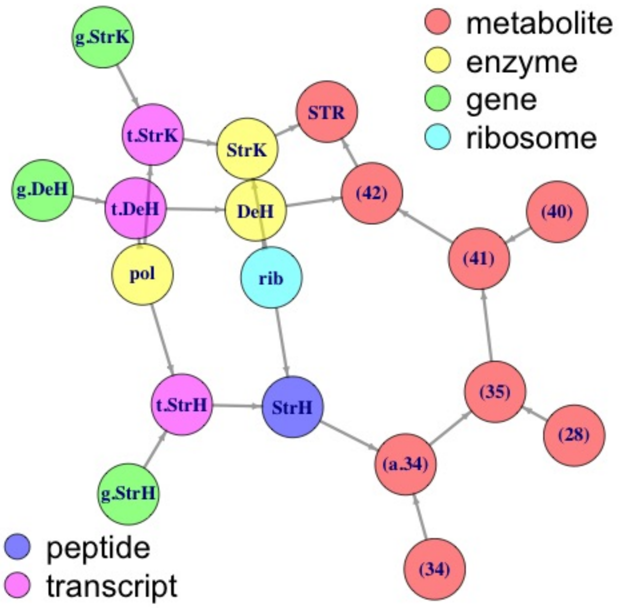

FIGURE 7. Streptomycin synthesis network with elements colored by type of element (for definitions of names see Table 5 in main text).

S1-8. Conclusions and perspectives

We have seen how the functions contained in our 'InterPlay' package help to classify the elements of a synthesis interactome (SI) as 'essential' or 'non essential', always in the framework of the information contained in the SI. We have also seen how plotting the networks among elements of the SI helps in the understanding of the information contained within them, reflecting essentiality of elements as cycles of synthesis.

We expect that the gathering of interactome information can be automatized from different data bases to have a comprehensive SI for the minimal cell, as proposed in the main text.

References

Bang-Jensen J and Gutin GZ (2008) *Digraphs: theory, algorithms and applications*. Springer Science & Business Media.

Berquist BR, DasSarma P, and DasSarma S (2007) Essential and non-essential dna replication genes in the model halophilic archaeon, halobacterium sp. nrc-1. *BMC genetics*, 8, 31.

Blomen VA, Májek P, Jae LT, Bigenzahn JW, Nieuwenhuis J, Staring J, Sacco R, van Diemen FR, Olk N, Stukalov A, et al. (2015) Gene essentiality and synthetic lethality in haploid human cells. *Science*, 350, 1092–1096.

Csardi G and Nepusz T (2006) The igraph software package for complex network research. *InterJournal*, Complex Systems, 1695. URL `http://igraph.org`.

Flatt PM and Mahmud T (2007) Biosynthesis of aminocyclitol-aminoglycoside antibiotics and related compounds. *Natural product reports*, 24, 358–392.

Gerdes S, Scholle M, Campbell J, Balazsi G, Ravasz E, Daugherty M, Somera A, Kyrpides N, Anderson I, Gelfand M, et al. (2003) Experimental determination and system level analysis of essential genes in escherichia coli mg1655. *Journal of bacteriology*, 185, 5673–5684.

Kobayashi K, Ehrlich SD, Albertini A, Amati G, Andersen K, Arnaud M, Asai K, Ashikaga S, Aymerich S, Bessieres P, et al. (2003) Essential bacillus subtilis genes. *Proceedings of the National Academy of Sciences*, 100, 4678–4683.

R Core Team (2016) *R: A Language and Environment for Statistical Computing.* R Foundation for Statistical Computing, Vienna, Austria. URL `https://www.R-project.org/`.

Schatz A, Bugle E, and Waksman SA (1944) Streptomycin, a substance exhibiting antibiotic activity against gram-positive and gram-negative bacteria.? *Proceedings of the Society for Experimental Biology and Medicine*, 55, 66–69.

Wang T, Birsoy K, Hughes NW, Krupczak KM, Post Y, Wei JJ, Lander ES, and Sabatini DM (2015) Identification and characterization of essential genes in the human genome. *Science*, 350, 1096–1101.
