# Supplementary material for: On an algorithmic definition for the components of the minimal cell
Source: PLoS One. 2018 Jun 1;13(6):e0198222. doi: 10.1371/journal.pone.0198222 (PMC5983409; doi:10.1371/journal.pone.0198222)
Supplement: S3 Text — Manual for our R package ‘InterPlay’ (included as ‘S1 Binary’). (PDF) [file pone.0198222.s003.pdf]

Package 'InterPlay'

February 2, 2018

Type Package

Title Obtains essential components in a synthesis interactome

Version 1.1

Date 2018-02-02

Author Octavio Martinez and M. Humberto Reyes-Valdes

Maintainer Octavio Martinez <octavio.martinez@cinvestav.mx>

Description A Synthesis Interactome (SI) is a data.frame with information for the synthesis of cell components. With this package SIs can be analyzed to algorithmically determine about the essentiality of cell components. Auxiliary functions for SIs analysis and examples of such structures are presented.

License GPL-3

R topics documented:

InterPlay-package 1
C2E 2
dummy1.SI 4
dummy2.SI 5
dummy3.SI 6
expandAll 7
findEssential 8
int.SI 9
normalize.SI 11
simulate.SI 12

Index **15**

`InterPlay-package` *Obtains essential components in a synthesis interactome*

Description

A Synthesis Interactome (SI) is a data.frame with information for the synthesis of cell components. With this package SIs can be analyzed to algorithmically determine about the essentiality of cell components. Auxiliary functions for SIs analysis and examples of such structures are presented.

Details

The DESCRIPTION file: This package was not yet installed at build time.

Index: This package was not yet installed at build time.
Knowledge about the synthesis of cell components can be summarized into a Synthesis Interactome (SI). Having a SI, we demonstrated that essential components are the ones that for their synthesis need their preexistence, i.e., essential components are recursive. Also, all elements that enter into the synthesis of an essential structure are essential (see references for details). With this package you can decide about the essentiality of components present in an SI. Various SI examples are presented, including one containing summarized information for the synthesis of the ribosome, the RNA polymerase and the metabolite streptomycin (`int.In`). From these examples we show how to classify each one of the elements as essential or nonessential.

Author(s)

Octavio Martinez and M. Humberto Reyes-Valdes

Maintainer: Octavio Martinez <octavio.martinez@cinvestav.mx>

References

Martinez Octavio and M. Humberto Reyes-Valdes. On an algorithmic definition for the components of the minimal cell. (in preparation).

Examples

```
# A small example without biological meaning:
data(dummy1.SI)
# Shows the raw data
dummy1.SI
# Normalize the data.set
normalize.SI(dummy1.SI)
# Find which elements are essential
findEssential(dummy1.SI)
# As above, but gives the sets to which each component belongs
findEssential(dummy1.SI, give.sets = TRUE)
```

`C2E` *Condensed to Expanded (C2E) algorithm*

Description

Beginning with a condensed formula with only two operands for a cell structure, the function obtains (when possible) an extended formula for the structure by performing nested substitutions on the condensed formula. If no extended formula is possible because the algorithm falls into an infinite loop, then the result is UNDECIDED.

Usage

```
C2E(s = "a", SI = dummy1.SI, sl = "<", sr = ">", no.spaces = TRUE, print.steps = FALSE, max.cycles =
```

Arguments

s	character string. The name of a component existent in the column name of the interactome SI.
SI	data.frame. An interactome with definitions for synthesis of cell structures.
sl	character string. A left-hand separator for the operands of a formula.
sr	character string. A right-hand separator for the operands of a formula.
no.spaces	logical. Are spaces to be ignored (deleted) in formulae? The default, code-TRUE, makes formulae more compact.
print.steps	logical. Are some intermediate steps of the C2E algorithm to be printed?
max.cycles	numeric. Maximum number of cycles of nested substitutions to be allowed before the algorithm exits.

Details

C2E will perform nested substitutions using the information of the interactome to expand the formula of the structure s. If the process is successful, the result will be the extended formula for s; however, if the algorithm falls into an infinite loop, then the result will be a list with the string "UNDECIDED" as first component and the second component will have a not fully expanded formula. The extent to which C2E perform nested substitutions is control by max.cycles.

Value

Either a character string giving the extended formula for component s, or a list with two components. See details.

Author(s)

Octavio Martinez

References

Martinez Octavio and M. Humberto Reyes-Valdes. On an algorithmic definition for the components of the minimal cell. (in preparation).

See Also

expandAll, findEssential, int.SI

Examples

```
# A small example
data(dummy1.SI)
# Find the expanded formula for component "a"
C2E(s="a", SI = dummy1.SI)
# Try to find the expanded formula for component "i"
# (but falls into an infinite loop and exit)
C2E(s="i", SI = dummy1.SI)
# Gives the formula with spaces
C2E(s="a", SI = dummy1.SI, no.spaces = FALSE)
# Uses different characters as operand separators
C2E(s="a", SI = dummy1.SI, sl = "[", sr = "]")
# Shows some of the steps of substitution
C2E(s="a", SI = dummy1.SI, print.steps = TRUE)
```

dummy1.SI *A dummy synthesis interactome with non biological meaning*

Description

A data.frame representing a synthesis interactome (SI) in which there is information for the synthesis of 5 structures. This example is intended to demonstrate the algebraic manipulations that the functions in the package can perform to determine which structures are essential and which are non essential. In total there are 9 structures mentioned within the SI, which names correspond to letters a to i.

Usage

```
data("dummy1.SI")
```

Format

A data frame with 5 observations on the following 3 variables.

- name A character vector with the names of structures defined in the SI (the set of internal structures Si).
- o1 A character vector with the left hand side operand of the binary operator for structure in column name.
- o2 A character vector with the right hand side operand of the binary operator for structure in column name.

References

Martinez Octavio and M. Humberto Reyes-Valdes. On an algorithmic definition for the components of the minimal cell. (in preparation).

Examples

```
data(dummy1.SI)
# Normalize the SI
normalize.SI(dummy1.SI)
# Normalize the SI giving also the sets of structures
normalize.SI(dummy1.SI, give.sets = TRUE)
# Obtain the expanded formulae
expandAll(dummy1.SI)
# See if there are essential structures in the SI
findEssential(dummy1.SI)
```

dummy2.SI *A dummy synthesis interactome (SI; example 2)*

Description

A `data.frame` representing a synthesis interactome (SI) in which there is information for the synthesis of 4 structures. This example is intended to demonstrate the algebraic manipulations that the functions in the package can perform to determine which structures are essential and which are non essential. In total there are 8 structures mentioned within the SI, which names correspond to letters `a` to `i`. This SI is augmented in `dummy3.SI`.

Usage

```
data("dummy2.SI")
```

Format

A data frame with 4 observations on the following 3 variables.

`name` A character vector with the names of structures defined in the SI (the set of internal structures `Si`).

`o1` A character vector with the left hand side operand of the binary operator for structure in column `name`.

`o2` A character vector with the right hand side operand of the binary operator for structure in column `name`.

References

Martinez Octavio and M. Humberto Reyes-Valdes. On an algorithmic definition for the components of the minimal cell. (in preparation).

Examples

```
data(dummy2.SI)
# Normalize the SI
normalize.SI(dummy2.SI)
# Normalize the SI giving also the sets of structures
normalize.SI(dummy2.SI, give.sets = TRUE)
# Obtain the expanded formulae
expandAll(dummy2.SI)
# See if there are essential structures in the SI
findEssential(dummy2.SI)
```

dummy3.SI *A dummy synthesis interactome (SI; example 3)*

Description

A `data.frame` representing a synthesis interactome (SI) in which there is information for the synthesis of 9 structures. This example grew from example 2 (`dummy2.SI`); see also that example. This example is complete and normalized; even has an extra column, `Comment`, which will not enter into the analyses and will be eliminated by `normalize.SI(dummy3.SI)`.

Usage

```
data("dummy3.SI")
```

Format

A data frame with 9 observations on the following 10 variables.

`name` A character vector with the names of structures defined in the SI (the set of internal structures `Si`).

`o1` A character vector with the left hand side operand of the binary operator for structure in column `name`.

`o2` A character vector with the right hand side operand of the binary operator for structure in column `name`.

`set.o1` a character vector with the name of the set to which `o1` belongs.

`set.o2` a character vector with the name of the set to which `o1` belongs.

`type.n` a character vector with the type of component that the structure on column `name` has.

`type.o1` a character vector with the type of component that the structure on column `o1` has.

`type.o2` a character vector with the type of component that the structure on column `o2` has.

`structure` a character vector with the name of the structure defined in the SI.

`Comment` a character vector with comments (this column will be eliminated by `normalize.SI(dummy3.SI)`). This, or further columns could be used to store extra information about the corresponding structures in column `name`.

Details

This dataset was augmented from `dummy2.SI` to show increased detail in the SI.

References

Martinez Octavio and M. Humberto Reyes-Valdes. On an algorithmic definition for the components of the minimal cell. (in preparation).

Examples

```
data(dummy3.SI)
# See the complete dataset
dummy3.SI
# Supress warnings
oldw <- getOption("warn")
options(warn = -1)
# normalize the dataset and put it in temp
temp <- normalize.SI(dummy3.SI)
# See and compare with original dummy3.SI
temp
# normalize the dataset including also the sets
temp2 <- normalize.SI(dummy3.SI, give.sets = TRUE)
# See the sets (second component of the output)
temp2$sets
# Find essential structures in this SI
# (you could also use temp2, the SI which includes sets)
findEssential(temp)
# Clean the mess and back the warn option to its state
# Clean the mess and back the warn option to its state
options(warn = oldw)
rm("temp", "temp2", "oldw")
```

`expandAll` *Expands all condensed formula for structures defined in a SI*

Description

This function uses the `C2E` algorithm to expand all the condensed formulae given in an synthesis interactome `SI`.

Usage

```
expandAll(SI = dummy1.SI, sl = "<", sr = ">", no.spaces = TRUE, max.cycles = 10)
```

Arguments

`SI`	`data.frame`. A syntesis interactome with definitions for synthesis of cell components.
`sl`	`character` string. A left-hand separator for the operands of a formula.
`sr`	`character` string. A right-hand separator for the operands of a formula.
`no.spaces`	`logical`. Are spaces to be ignored (deleted) in formulae? The default, code-TRUE, makes formulae more compact.
`max.cycles`	`numeric`. Maximum number of cycles of nested substitutions to be allowed before the `C2E` algoritm exits.

Details

If the expanded formula for a given structure cannot be found, the result for that formula is UNDECIDED.

Value

A data.frame with columns name, condensed and expanded, containing, for each row of the input synthesis interactome, SI, the name of the component, and its condensed and expanded formulae, respectively.

Author(s)

Octavio Martinez

References

Martinez Octavio and M. Humberto Reyes-Valdes. On an algorithmic definition for the components of the minimal cell. (in preparation).

See Also

C2E

Examples

```
data(dummy1.SI)
# Obtains all expanded formula for the structures defined in dummy1.SI
expandAll(SI = dummy1.SI)
# Using some cosmetic options
expandAll(SI = dummy1.SI, sl="[", sr="]", no.spaces = FALSE)
```

---

findEssential   *Find essential components from a synthesis interactome (SI)*

Description

findEssential, qualify each structure as essential or non essential given the information of an SI. Essentiality rules are: 1) A component is essential if it needs their own existence for its synthesis and 2) A component is essential if it enters in the synthesis of an essential component. This is the main function of this package.

Usage

```
findEssential(SI = dummy1.SI, operand.joiner = ";", give.sets = FALSE, give.Expanded = TRUE, sl = '
```

Arguments

SI	data.frame. An synthesis interactome with definitions for the synthesis of some cell components.
operand.joiner	character string. A character to separate operands in the output.
give.sets	logical. Will the sets of structures be part of the output?
give.Expanded	logical. Will the expanded formulae be part of the output?
sl	character. A left-hand separator for the operands of a formula.
sr	character. A right-hand separator for the operands of a formula.

Details

The function will find all operands that enter into the synthesis of each component defined in the SI (all structures in column `name` of the SI). To do this it performs nested substitutions of binary operators. However components which are found to be recursive (and thus are essential) are 'frozen' and no further substitution is performed when they appear as operands. This avoids infinite loops within the substitution chain. Additionally, the function will qualify as essential all operators which are found to be needed for the synthesis of recursive structures.

Value

Either a `data.frame`, when `give.sets = FALSE` or a list of two components, `main` and `sets` when `give.sets = FALSE`.

`main`	A `data.frame` with main results of the algorithm (This is the only component returned when `give.sets = FALSE`)
`sets`	A `list` with the sets in which each component was classified

Author(s)

Octavio Martinez and M. Humberto Reyes-Valdes

References

Martinez Octavio and M. Humberto Reyes-Valdes. On an algorithmic definition for the components of the minimal cell. (in preparation).

See Also

`C2E`, `expandAll`, `int.SI`

Examples

```
data(dummy1.SI)
# See original data
dummy1.SI
# Perform analysis to find essential structures
# with the default parameters
findEssential(SI = dummy1.SI)
# and asking for the sets in which the components are classified
findEssential(SI = dummy1.SI, give.sets = TRUE)
# Using some cosmetic options
findEssential(SI = dummy1.SI, operand.joiner=":", sl="[", sr="]")
```

`int.SI` *Example of an integrated synthesis interactome (SI)*

Description

This interactome has simplified information for the synthesis of RNA polymerase (`pol`), the ribosome (`rib`) and the secondary metabolite streptomycin (`STR`). Such information is given as a list of binary operators which symbolizes the molecular binding that happens between pairs of structures from which a new structure is synthesized.

Usage

```
data("int.SI")
```

Format

A data frame with 184 observations on the following 9 variables.

`name` a character vector. Name of structures in the set of internal structures, `Si`.

`o1` a character vector. Left hand side operand of the binary operator for structure in column `name`.

`o2` a character vector. Right hand side operand of the binary operator for structure in column `name`.

`set.o1` a character vector with the name of the set to which `o1` belongs.

`set.o2` a character vector with the name of the set to which `o1` belongs.

`type.n` a character vector with the type of component that the structure on column `name` has.

`type.o1` a character vector with the type of component that the structure on column `o1` has.

`type.o2` a character vector with the type of component that the structure on column `o2` has.

`structure` a character vector with the name of the structures defined in the SI (in this case it has 3 different values).

Details

Simplified information for the synthesis of RNA polymerase (`pol`), the ribosome (`rib`) and the secondary metabolite streptomycin (`STR`; see source) was compiled from the literature and expressed as a set of binary operators with the aim of illustrating the algebraic manipulations that can be performed to segregate essential from non essential cell structures. Different and more detailed representations of the synthesis of that structures are possible, but the ones given here are biologically realistic and allow the presentation of the main results (see reference).

Source

Streptomycin synthesis simplified from: Flatt PM, Mahmud T. Biosynthesis of aminocyclitol aminoglycoside antibiotics and related compounds. Natural product reports. 2007;24(2):358 to 392.

References

Martinez Octavio and M. Humberto Reyes-Valdes. On an algorithmic definition for the components of the minimal cell. (in preparation).

Examples

```
data(int.SI)
# This SI do not contains sets (yet).
# See the first two rows of the SI
head(int.SI, n = 2)

# See which structures are defined in the Si
unique(int.SI$structure)
# Note: "rib" = ribosome, "pol" = RNA polymerase, "STR" = streptomycin.
# How many rows do we have
nrow(int.SI)
# Let's normalize the SI and get the sets
temp <- normalize.SI(int.SI, give.sets = TRUE)
names(temp)
```

```
# Note: "SI" - main SI; "sets" - Structures within the SI by set
names(temp$sets)
# Note: "G" - Genomic elements, "Si" - Internal elements, "Se" - External elements.
# See the set Se of elements not defined within the SI:
temp$sets$Se
# Isolate independent sets for the synthesis of
# RNA polymerase (pol) and streptomycin (STR)
temp.pol <- int.SI[int.SI$structure == "pol",]
temp.STR <- int.SI[int.SI$structure == "STR",]

# Use findEssential to determine essential structures
# within these (partial) interactomes
findEssential(temp.pol)
findEssential(temp.STR)

# Clean the mess
rm(temp, temp.pol, temp.STR)
```

`normalize.SI` *Check and normalizes a synthesis interactome (SI)*

Description

In this package SIs are represented in a `data.frame` format with three main columns: `name` - The name of the components and `o1`, `o2` the operands of the binary operator from which `name` is synthesized. Synthesis of any cell component can be represented in an SI up to any degree of detail. This function checks that the input is a well formed SI, adding or deleting columns and assigning the sets to which each one of the components belong. Those sets are: `Si` - the set of internal components which synthesis is defined in the SI, `Se` - The set of external structures and `G` - the set of genomic elements (genes, DNA motifs, etc.). This function re-assign (if necessary) the structures to the sets, in agreement with the information of the SI and optionally a list with the sets.

Usage

```
normalize.SI(SI = dummy1.SI, give.sets = FALSE)
```

Arguments

`SI`	A putative synthesis interactome (SI) with definitions for synthesis of cell components.
`give.sets`	A logical (`TRUE` or `FALSE`) variable to indicate if the output will contain sets or not.

Details

Other functions in this package use normalized interactomes; i.e., if the input SI is not normalized, that is the first step performed. Normalization consist on checking the structure of the input SI, adding or deleting columns. Rules for well formed SI are checked, and, if not passed the function gives an error explaining the problem. Also `Warnings` could be obtained when changes are performed to the input to perform normalization.

Value

Either a `data.frame`, (containing the `SI`) when `give.sets = FALSE` or a list with two components, `SI` and `sets` when `give.sets = TRUE`.

`SI`	A normalized SI with nine columns and as many rows as the input.
`sets`	A list with the sets; i.e., with components:
`G`	Names of structures in the set of genomic elements.
`Si`	Names of structures in the internal structures set.
`Se`	Names of structures in the external structures set.

Author(s)

Octavio Martinez.

References

Martinez Octavio and M. Humberto Reyes-Valdes. On an algorithmic definition for the components of the minimal cell. (in preparation).

See Also

`dummy1.SI`, `int.SI`

Examples

```
# Minimal example with a dummy interactome:
data(dummy1.SI)
# See the data
dummy1.SI
# Normalize this SI without obtaining the sets:
normalize.SI(dummy1.SI)
# Normalize this SI obtaining the sets:
normalize.SI(dummy1.SI, give.sets = TRUE)
# Obtains a random SI
temp <- simulate.SI()
temp # Look at the data
# Normalize this SI
normalize.SI(temp)
# Erase the result
rm(temp)
```

`simulate.SI` *Simulates a pseudo-random synthesis interactome (SI)*

Description

Simulates an small and random SI to be analyzed by other functions.

Usage

```
simulate.SI(n.S = 10, n.Si = 5, seed = NULL)
```

Arguments

`n.S`	`integer` < 27. The maximum number of structures to be included in the SI.
`n.Si`	`integer <= n.S`. The number of structures to be defined in the SI.
`seed`	A seed for the pseudo random generator (optional).

Details

The function will always give well formed (random) SIs.

Value

A (random) synthesis interactome (SI)

Author(s)

Octavio Martinez.

References

Martinez Octavio and M. Humberto Reyes-Valdes. On an algorithmic definition for the components of the minimal cell. (in preparation).

See Also

`C2E`, `expandAll`, `findEssential`

Examples

```
# Testing with defaults
simulate.SI()
# A tiny SI with high probability of having an essential structure
temp <- simulate.SI(n.S=3, n.Si=3)
# See your creation
temp
# See if it has essential structures
findEssential(temp)
# Clean the mess
rm(temp)
# A SI with a low probability of having essential structures
temp <- simulate.SI(n.S=26, n.Si=3)
# See your creation
temp
# See if it has essential structures
findEssential(temp)
# Clean the mess
rm(temp)
# If you want to be able to reproduce use a specific seed
temp <- simulate.SI(n.S = 10, n.Si = 5, seed = 1959)
# See the results
temp
# Repeat
simulate.SI(n.S = 10, n.Si = 5, seed = 1959)
# Play
expandAll(temp)
findEssential(temp)
```

```
# Clean the mess
rm(temp)
```

Index

∗Topic **datasets**
dummy1.SI, 4
dummy2.SI, 5
dummy3.SI, 6
int.SI, 9

C2E, 2, *8*, *9*, *13*

dummy1.SI, 4, *12*
dummy2.SI, 5
dummy3.SI, 6

expandAll, *3*, 7, *9*, *13*

findEssential, *3*, 8, *13*

int.SI, *3*, *9*, 9, *12*
InterPlay *(*InterPlay-package*)*, 1
InterPlay-package, 1

normalize.SI, 11

simulate.SI, 12
